# Cueing memory reactivation during NREM sleep engenders long-term plasticity in both brain and behaviour

**DOI:** 10.1162/imag_a_00250

**Published:** 2024-08-01

**Authors:** Martyna Rakowska, Paulina Bagrowska, Alberto Lazari, Miguel Navarrete, Mahmoud E. A. Abdellahi, Heidi Johansen-Berg, Penelope A. Lewis

**Affiliations:** Cardiff University Brain Research Imaging Centre (CUBRIC), School of Psychology, Cardiff University, Cardiff, United Kingdom; Experimental Psychopathology Lab, Institute of Psychology, Polish Academy of Sciences, Warsaw, Poland; Wellcome Centre for Integrative Neuroimaging, FMRIB, Nuffield Department of Clinical Neurosciences, University of Oxford, Oxford, United Kingdom; Department of Biomedical Engineering, Universidad de los Andes, Bogotá, Colombia

**Keywords:** sleep, MRI, TMR, plasticity, EEG, memory

## Abstract

Memory reactivation during Non-Rapid Eye Movement (NREM) sleep is important for memory consolidation but it remains unclear exactly how such activity promotes the development of a stable memory representation. We used Targeted Memory Reactivation (TMR) in combination with longitudinal structural and functional MRI to track the impact of reactivating memories in one night of sleep over the next 20 days. Our exploratory analysis showed that such cued reactivation leads to increased precuneus activation 24 h post-TMR. Furthermore, the behavioural impact of cueing, which only emerged 20 days later, was predicted by both functional and structural TMR related changes in the sensorimotor cortex. These preliminary findings demonstrate that TMR leads to neuroplasticity, starting as early as 24 h after the manipulation, and evolving over the next few weeks.

## 1 Introduction

Memory consolidation is a process through which newly encoded memories become more stable and long-lasting. Consolidation is thought to involve repeated reinstatement, or reactivation of memory traces which allows their re-coding from short-term to long-term store (McClelland et al., 1995). Reactivation of learning-related brain activity patterns during sleep has been shown to predict subsequent memory performance (Deuker et al., 2013;Peigneux et al., 2004) and thus to play a critical role in memory consolidation (Born & Wilhelm, 2012;Diekelmann & Born, 2010). However, it is unclear exactly how such offline rehearsal promotes the development of a stable memory representation. Here, we set out to investigate the neuroplasticity underlying memory reactivation during sleep using Targeted Memory Reactivation (TMR) and magnetic resonance imaging (MRI).

TMR has recently emerged as a tool to study memory reactivation. This technique involves re-presenting learning-associated cues during sleep (Rasch et al., 2007), thereby triggering reactivation of the associated memory representation and biasing their consolidation (Bendor & Wilson, 2012). In humans, this manipulation leads to strong behavioural effects (Antony et al., 2012;Cousins et al., 2016;Rakowska et al., 2021;Schönauer et al., 2014), resulting in better recall of memories that were cued through TMR compared to those that were not cued. Functional activity associated with cueing has been investigated during and immediately after sleep (Cousins et al., 2016;Rasch et al., 2007;Shanahan et al., 2018;van Dongen et al., 2012). However, little is known about precisely how the memory representations targeted by TMR evolve over longer time periods. We have previously reported behavioural effects of memory cueing during sleep 20 days post-manipulation (Rakowska et al., 2021). Yet, the functional plasticity underlying such benefits is unknown. Furthermore, whether TMR can impact on brain structure and which regions support sleep-dependent memory consolidation in the long term remain to be established.

**Fig. 1.**
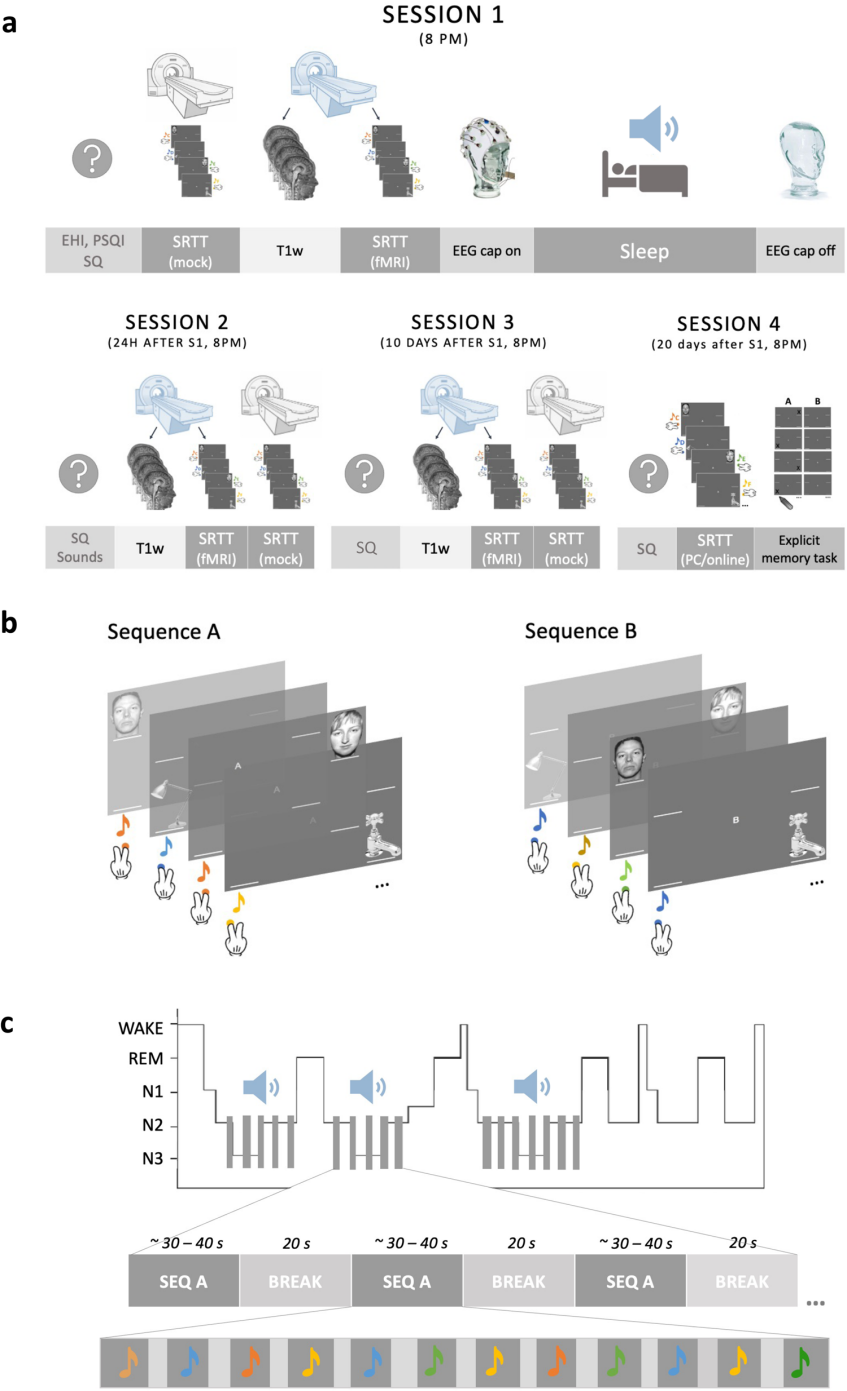
Study design and methods. (a) A schematic representation of the experimental sessions. SRTT and one or more questionnaires were delivered in each session. During S1–S3, SRTT was split in half, with the first half completed in the 0T “mock” scanner (to acclimate subjects to the scanner environment) (grey) and the second half in the 3T MRI scanner during fMRI acquisition (blue) (S1), or vice versa (S2–S3). T1w data were always acquired before fMRI. S1 also involved a stimulation night in the lab which the participants spent asleep and with the electroencephalography (EEG) cap on. During S4, SRTT data were acquired outside the MRI scanner and an explicit memory task was delivered at the very end of the study (seeFig. S4for results). (b) Two sequences of the SRTT. Only the first few trials are shown. Visual cues appeared at the same time as the auditory cues, and the participants were instructed to push the key/button corresponding to the image location as quickly and accurately as possible. (c) TMR protocol. Tones associated with one sequence were played during stable N3 and N2 (grey bars on the hypnogram). One repetition of the cued sequence (dark grey rectangles) was followed by a 20 s break during which no sounds were played (light grey rectangles). Each sequence repetition comprised 12 tones (depicted as coloured notes) with inter-trial interval jittered between 2,500 and 3,500 ms (light grey vertical bars). S1–S4: Session 1–Session 4; EHI: Edinburgh Handedness Inventory; PSQI: Pittsburgh Sleep Quality Index; SQ: Stanford Sleepiness Scale Questionnaire; SRTT: Serial Reaction Time Task; fMRI: functional Magnetic Resonance Imaging; T1w: T1-weighted scan.

In this study, we used TMR to determine if repeated reactivation of a memory trace during sleep engenders learning-related changes in the brain. We tracked such impacts over several weeks using both functional and structural brain imaging (Fig. 1a) and hypothesised that memory cueing during sleep would lead to rapid plasticity within the precuneus, a structure which houses newly formed memory representations or “engrams” (Brodt et al., 2018). This region was of special interest since it has been shown to respond to repeated learning-retrieval epochs which help to strengthen a memory (Brodt et al., 2018) and can be thought of as a proxy for memory reactivation in sleep (Himmer et al., 2019).

We chose to focus specifically on a Serial Reaction Time Task (SRTT) because the importance of sleep in motor sequence learning is well established (Loganathan, 2014;Walker, 2005). Furthermore, improvements on motor tasks (Walker et al., 2003) and the associated structural changes (Kodama et al., 2018) have been shown to persist over time, with the same being true for the TMR effects (Rakowska et al., 2021). Our participants were trained on a Serial Reaction Time Task (SRTT), learning two motor sequences of 12-item button presses. Each sequence was associated with a different set of auditory tones (Fig. 1b) but only one was reactivated during subsequent NREM sleep (Fig. 1c). During learning and two post-sleep re-test sessions (24 h and 10 days post-TMR), participants were scanned with structural MRI (T1-weighted) and functional MRI (fMRI) acquired during SRTT performance. We were thus able to perform exploratory analysis and compare brain activity during the cued and uncued sequence performance, as well as scrutinising brain structure after the first 10 days post-stimulation. Twenty days post-TMR, participants were again re-tested on the SRTT, now outside the scanner (online testing at home), allowing us to examine the long-term impacts of TMR on behaviour and relate this to functional and structural changes in the brain. The resultant dataset enabled us to investigate when the behavioural impacts of cueing emerge, and to study the relationships between structural, functional, and behavioural plasticity post-TMR. Importantly, while we were interested in the precuneus as a putative seat for the “engram,” we also expected the long-term storage of the memory engram to prevail in strongly task-related areas that are known to respond to TMR such as the hippocampus, striatum, and cerebellum (Cousins et al., 2016). Additionally, the sensorimotor cortex is so clearly necessary for this task that we expected responses there.

## 2 Methods

### 2.1 Participants

A pre-study questionnaire was used to exclude subjects with a history of drug/alcohol abuse, psychological, neurological, or sleep disorders, hearing impairments, recent stressful life event(s), or regular use of any medication or substance affecting sleep. Participants were required to be right-handed, non-smokers, have regular sleep patterns, normal or corrected-to-normal vision, no prior knowledge of the tasks used in the study, and no more than 3 years of musical training in the past 5 years as musical training has previously been shown to affect procedural learning (Romano Bergstrom et al., 2012). None of the participants reported napping regularly, working night shifts or travelling across more than two time-zones 1 month prior to the experiment. Thirty-three volunteers fulfilled all inclusion criteria and provided informed consent to participate in the study, which was approved by the Ethics Committee of the School of Psychology at Cardiff University (ethics number EC.19.06.11.5651R3A2) and performed in accordance with the Declaration of Helsinki. All participants agreed to abstain from extreme physical exercise, napping, alcohol, caffeine, and other psychologically active food from 24 h prior to each experimental session. Finally, before their first session, participants were screened by a qualified radiographer from Cardiff University to assess their suitability for MRI and signed an MRI screening form prior to each scan.

Three participants had to be excluded from all analyses due to: technical issues (n = 1), voluntary withdrawal (n = 1), and low score on the handedness questionnaire (indicating mixed use of both hands), combined with a positive slope of learning curve during the first session (indicating lack of sequence learning before sleep) (n = 1). Hence, the final dataset included 30 participants (16 females, age range: 18–23 years, mean ± SD: 20.38 ± 1.41; 14 males, age range: 19–23 years, mean ± SD: 20.43 ± 1.16). However, due to the COVID-19 outbreak, six participants were unable to complete the study, missing all data from either one (n = 1) or two (n = 5) sessions. Hence, n = 25 for all data collected during S3 and n = 24 for S4. The final dataset included one participant who could not physically attend S3. They performed the SRTT online, but their MRI data (functional, fMRI and structural, T1w) could not be collected and therefore the sample size for the MRI analyses of S3 had to be further decreased by one. Two additional participants were excluded from the fMRI analysis of S2 due to MRI gradient coil damage during fMRI acquisition (n = 1) and failure to save the fMRI data (n = 1). Hence, the final sample size for fMRI was n = 30 for S1, n = 28 for S2 and n = 24 for S3, whereas the final sample size for analysis of T1w data was n = 30 for S1, n = 30 for S2, and n = 24 for S3. Finally, one participant had to be excluded from all the analyses concerning EEG due to substantial loss of data caused by failure of the wireless amplifier during the night. However, the TMR procedure itself was unaffected and therefore this participant was included in the behavioural and MRI analyses.

### 2.2 Experimental design

The experiment consisted of four sessions (Fig. 1a), all scheduled for ~8 pm. Upon arrival for the first session, participants completed Pittsburgh Sleep Quality Index (PSQI) (Buysse et al., 1989) to examine their sleep quality over the past month and Stanford Sleepiness Questionnaire (SQ) (Hoddes et al., 1973) to assess their current level of alertness. A short version of the Edinburgh Handedness Inventory (Veale, 2014) was also administered to confirm that all subjects were right-handed before the learning session took place. Due to time constraints at the MRI scanner, the learning session had to be split into two parts. The first half of the SRTT blocks (24 sequence blocks) was performed in a 0T Siemens “mock” scanner which also helped to acclimate subjects to the scanner environment. The second half of the SRTT blocks (24 sequence blocks + 4 random blocks) was performed in a 3T Siemens MRI scanner during fMRI acquisition and used for functional data analysis. fMRI acquisition was preceded by a structural scan (T1w) and followed by a B0 fieldmap (seesection 2.6*MRI data acquisition*). Once outside the MRI scanner, participants were asked to prepare themselves for bed. They were fitted with an EEG cap and were ready for bed at ~11 pm. During N2 and N3 sleep stages, tones associated with one of the SRTT sequences were replayed to the participants via speakers (Harman/Kardon HK206, Harman/Kardon, Woodbury, NY, USA) to trigger reactivation of the SRTT memories associated with them. Participants were woken up after, on average, 8.81 ± 0.82 h in bed and had the EEG cap removed before leaving the lab.

We asked participants to come back for the follow-up sessions 23–26 h (session 2, S2), 10–14 days (session 3, S3), and 16–21 days (session 4, S4) after S1. The choice of 16–21 days as the final time point was deliberate, guided by our previous findings, which demonstrated a TMR effect at day 10 post-stimulation but not 6 weeks later. All the follow-up, sessions were scheduled for the same time in the evening to control for the time-of-day effect observed in MRI data (Trefler et al., 2016). During S2, participants were asked to indicate if they remember hearing any sounds during the night in the lab. S2 and S3 lasted ~2 h each and both involved the SQ and an MRI scan, during which a structural scan was acquired. This was followed by the SRTT re-test, with the first half of the SRTT blocks (24 sequence blocks + 4 random blocks) performed during the fMRI acquisition and the second half (24 sequence blocks + 4 random blocks) in the mock scanner. Note that the order of scanners (3T vs. 0T) was flipped from S1 to S2 and S3 for the functional and structural assessment to occur as close to the TMR session as possible. S4 took place either in the lab or online, depending on the severity of COVID-19 restrictions at the time. During S4, SQ was delivered as before, together with the SRTT (one run, 48 sequence blocks + 4 random blocks) and an explicit memory task. Upon completion of each session, participants were informed about the upcoming SRTT re-tests as this has been shown to enhance post-learning sleep benefits (Wilhelm et al., 2011).

For offline data collection, the SRTT (S1–S3) was back projected onto a projection screen situated at the end of the MRI/mock scanner and reflected into the participant’s eyes via a mirror mounted on the head coil; the questionnaires and the SRTT (S4) were presented on a computer screen with resolution 1,920 x 1,080 pixels, and the explicit memory task was completed with pen and paper. SRTT was presented using MATLAB 2016b (The MathWorks Inc., Natick, MA, USA) and Cogent 2000 (developed by the Cogent 2000 team at the Functional Imaging Laboratory and the Institute for Cognitive Neuroscience, University College, London, UK;http://www.vislab.ucl.ac.uk/cogent.php); questionnaires were presented using MATLAB 2016b and Psychophysics Toolbox Version 3 (Brainard & Vision, 1997).

For online data collection, SRTT (S4) was coded in Python using PsychoPy 3.2.2. (Peirce et al., 2019) and administered through the Pavlovia online platform (https://pavlovia.org/); questionnaires were distributed via Qualtrics software (Qualtrics, 2005), and the explicit memory task was sent to the participants as a .pdf document which they were asked to edit according to the instructions provided.

### 2.3 Experimental tasks

#### 2.3.1 Motor sequence learning—the serial reaction time task (SRTT)

The SRTT (Fig. 1b) was used to induce and measure motor sequence learning. It was adapted from (Cousins et al., 2014), as described previously (Rakowska et al., 2021). SRTT consists of two 12-item sequences of auditorily and visually cued key presses, learned by the participants in blocks. The task was to respond to the stimuli as quickly and accurately as possible, using index and middle fingers of both hands. The two sequences—A (1–2–1–4–2–3–4–1–3–2–4–3) and B (2–4–3–2–3–1–4–2–3–1–4–1)—were matched for learning difficulty, they did not share strings of more than four items and contained items that were equally represented (three repetitions of each). Each sequence was paired with a set of 200 ms-long tones, either high (5^th^octave, A/B/C#/D) or low (4^th^octave, C/D/E/F) pitched, that were counterbalanced across sequences and participants. For each item/trial, the tone was played with simultaneous presentation of a visual cue in one of the four corners of the screen. Visual cues consisted of neutral faces and objects appearing in the same location regardless of the sequences (1—top left corner = male face, 2—bottom left corner = lamp, 3—top right corner = female face, 4—bottom right corner = water tap). Participants were told that the nature of the stimuli (faces/objects) was not relevant for the study. Their task was to press the key on the keyboard (while in the sleep lab or at home) or on an MRI-compatible button pad (2-Hand system, NatA technologies, Coquitlam, Canada) (while in the MRI/mock scanner) that corresponded to the position of the picture as quickly and accurately as possible: 1 = left shift/left middle finger button; 2 = left Ctrl/left index finger button; 3 = up arrow/right middle finger button; 4 = down arrow/right index finger button. Participants were instructed to use both hands and always keep the same fingers on the appropriate response keys. The visual cue disappeared from the screen only after the correct key was pressed, followed by a 300 ms interval before the next trial.

There were 24 blocks of each sequence (a total of 48 sequence blocks per session). The block type was indicated with “A” or “B” displayed in the centre of the screen. Each block contained three sequence repetitions (36 items) and was followed by a 15 s pause/break, with reaction time and error rate feedback. Blocks were interleaved pseudo-randomly with no more than two blocks of the same sequence in a row. Participants were aware that there were two sequences but were not asked to learn them explicitly. Block order and sequence replayed were counterbalanced across participants.

During each run of the SRTT, sequence blocks A and B were followed by 4 random blocks except for in the first half of S1 (to avoid interrupting learning, most of which occurred during S1). Random blocks were indicated with “R” appearing in the centre of the screen and contained pseudo-randomised sequences. For these, visual stimuli were the same and tones matched sequence A tones for half of them (Rand_A) and sequence B tones for the other half (Rand_B). Blocks Rand_A and Rand_B were alternated, and each contained random sequences constrained by the following criteria: 1) cues within a string of 12 items were equally represented, 2) the same cue did not occur in consecutive trials, and 3) the sequence did not share more than four cues in a row with either sequence A or B.

#### 2.3.2 Explicit memory task

Explicit memory of the SRTT was assessed by a free recall test administered at the end of the study (S4). Participants were provided with printed screenshots of sequence A and sequence B trials, but the visual cues were removed. They were instructed to mark the order of each sequence by drawing an “X” to indicate cue location.

### 2.4 EEG data acquisition

EEG data were acquired with actiCap slim active electrodes (Brain Products GmbH, Gilching, Germany). Sixty-two scalp electrodes were embedded within an elastic cap (Easycap GmbH, Herrsching, Germany), with the reference electrode positioned at CPz and ground at AFz. Electromyogram (EMG) signals were recorded from two electrodes placed on the chin, whereas the electrooculogram (EOG) was collected from two electrodes placed below the left eye and above the right eye. Elefix EEG-electrode paste (Nihon Kohden, Tokyo, Japan) was applied on each electrode for stable attachment, and Super-Visc high viscosity electrolyte gel (Easycap GmbH) was used to keep impedance below 25 kOhm. Signals were amplified with either two BrainAmp MR plus EEG amplifiers or LiveAmp wireless amplifiers and recorded using BrainVision Recorder software (all from Brain Products GmbH).

### 2.5 TMR during NREM sleep

The TMR protocol was administered as in our prior study (Rakowska et al., 2021), using MATLAB 2016b and Cogent 2000. Briefly, tones associated with either sequence A or B (counterbalanced across participants) were replayed to the participants during stable N2 and N3 (Fig. 1c) irrespective of slow wave phase or spindle occurrence. Presentation of sounds during sleep was manually controlled by the experimenters, who initiated TMR when the target sleep stage was identified and paused it when participants exhibited signs of arousal or shifted to a non-target sleep-stage. Replay blocks contained one repetition of a sequence (i.e., 12 sounds) and were followed by 20 s of silence. The inter-trial interval between individual sounds was jittered between 2,500 and 3,500 ms. Volume was adjusted manually for each participant to prevent arousal. However, upon leaving the relevant sleep stage, replay was paused and resumed only when stable N2 or N3 was observed. TMR was performed until ~1,000 trials were delivered in N3. On average, 1,385.20 ± 305.53 sounds were played.

### 2.6 MRI data acquisition

Magnetic resonance imaging (MRI) was performed at Cardiff University Brain Imaging Centre (CUBRIC) with a 3T Siemens Connectom scanner (maximum gradient strength 300 mT/m). All scans were acquired with a 32-channel head-coil and lasted ~1 h in total each, with whole-brain coverage. Apart from the T1w and fMRI scans, the MRI protocol also included multi-shell Diffusion-Weighted Imaging (DWI) and mcDESPOT acquisitions, but these are not discussed here.

#### 2.6.1 T1-weighted imaging

A high-resolution T1w anatomical scan was acquired with a 3D magnetization-prepared rapid gradient echoes (MPRAGE) sequence (2,300 ms repetition time [TR]; 2 ms echo time [TE]; 857 ms inversion time [TI]; 9° flip angle [FA]; bandwidth 230 Hz/Pixel; 256 mm field-of-view [FOV]; 256 x 256 voxel matrix size; 1 mm isotropic voxel size; 1 mm slice thickness; 192 sagittal slices; parallel acquisition technique [PAT] with in-plane acceleration factor 2 (GRAPPA); anterior-to-posterior phase-encoding direction; 5 min total acquisition time [AT]) at the beginning of each scanning session.

#### 2.6.2 Functional MRI

Functional data were acquired with a T2*-weighted multi-band echo-planar imaging (EPI) sequence (2,000 ms TR; 35 ms TE; 75° FA; bandwidth 1,976 Hz/Pixel; 220 mm FOV; 220 x 220 voxel matrix size; 2 mm isotropic voxel size; 2 mm slice thickness; 87 slices with a ~25° axial-to-coronal tilt from the anterior–posterior commissure (AC-PC) line and interleaved slice acquisition; PAT 2 (GRAPPA); multi-band acceleration factor [MB] 3; anterior-to-posterior phase-encoding direction; maximum 24 min AT and 720 scans; because the task was self-paced the exact AT and the number of scans differed between participants). Each fMRI acquisition was preceded by dummy scans to allow for saturation of the MR signal before the start of the task. Due to the nature of the task, the fMRI paradigm followed a block design consisting of sequence and random blocks (self-paced), alternating with rest blocks (15 s) (seesection 2.3.1*Motor sequence learning*—*the serial reaction time task (SRTT)*). Presentation of the first stimulus in a block was synchronised with the scanner’s trigger signal sent upon acquisition of every fMRI volume. Thus, the beginning of the task (i.e., the first stimulus of the first block) was triggered by the first fMRI volume acquisition and for that reason the initial volumes did not have to be discarded. No online motion correction was applied.

#### 2.6.3 B0 fieldmap

B0-fieldmap was acquired to correct for distortions in the fMRI data caused by magnetic field (i.e., B0) inhomogeneities (465 ms TR; 4.92 ms TE; 60° FA; bandwidth 290 Hz/Pixel; 192 mm FOV; 192 x 192 voxel matrix size; 3 mm isotropic voxel size; 3 mm slice thickness; 44 slices with a ~25° axial-to-coronal tilt from the AC-PC line and interleaved slice acquisition; 1 average; anterior-to-posterior phase-encoding direction; 1 min AT).

### 2.7 Data analysis

#### 2.7.1 Behavioural data

##### 2.7.1.1 SRTT: Reaction time

SRTT performance was measured using mean reaction time per block of each sequence (cued and uncued). Both hands (BH) dataset contained all SRTT trials within each block, except for those with reaction time exceeding 1,000 ms. Trials with incorrect button presses prior to the correct ones were included in the analysis. All analysis reported in-text concerns trials performed with both hands. However, given our previous results on this task (Koopman et al., 2020;Rakowska et al., 2021), we were also interested in unpacking the effects of cueing on the SRTT performance of each hand separately. To this end, the BH dataset was divided into the right hand (RH) dataset and left hand (LH) dataset, where each contained only the trials performed with the dominant or non-dominant hand, respectively. For each sequence within a given dataset, the mean performance on the 4 target blocks was subtracted from the mean performance on the 2 random blocks. This allowed us to separate sequence learning from sensorimotor mapping and thus obtain a measure of “sequence-specific skill” (SeqSpecS). The target blocks were the first 4 sequence blocks, used to calculate early SeqSpecS, and the last 4 sequence blocks, used to calculate late SeqSpecS, as illustrated below:

1. Early SeqSpecS = mean (random blocks) – mean (first 4 sequence blocks)
2. Late SeqSpecS = mean (random blocks) – mean (last 4 sequence blocks)

Finally, to obtain a single measure reflecting the effect of TMR on the SRTT performance we calculated the difference between the SeqSpecS of the cued and uncued sequence and refer to it as the “cueing benefit.”

##### 2.7.1.2 Questionnaires

PSQI global scores were determined in accordance with the original scoring system (Buysse et al., 1989). Answers to the short version of the EHI were scored as in (Veale, 2014) and used to obtain laterality quotient for handedness. For results, seeSupplementary Notes: Questionnaires.

##### 2.7.1.3 Explicit memory task

Responses on the explicit memory task were considered correct only if they were in the correct position within the sequence and next to at least one other correct item, hence reducing the probability of guessing (Cousins et al., 2014). The number of items guessed by chance was determined for each participant by taking an average score of 10 randomly generated sequences. To test if the explicit memory was formed, the average chance level across all participants was compared with the average number of correct items for each sequence. For results, see Supplementary Notes: Explicit Memory Task andFigure S4.

#### 2.7.2 EEG data analysis

All EEG data were analysed in MATLAB 2018b using FieldTrip Toolbox (Oostenveld et al., 2011).

##### 2.7.2.1 Sleep scoring

EEG signal was recorded throughout the night at eight scalp electrodes (F3, F4, C3, C4, P3, P4, O1, O2); two EOG and two EMG channels were pre-processed and re-referenced from CPz to the mastoids (TP9, TP10). For two participants, the right mastoid channel (TP10) was deemed noisy through visual inspection and had to be interpolated based on its triangulation-based neighbours (TP8, T8, P8), before it could be used as a new reference. The data were scored according to the AASM criteria (Berry et al., 2015) by two independent sleep scorers who were blinded to the cue presentation periods. Any disagreements between the scorers were resolved through discussion. Sleep scoring was performed using a custom-made interface (https://github.com/mnavarretem/psgScore).

##### 2.7.2.2 Spindles analysis

The relationship between sleep spindles and behavioural measures was assessed using 8 electrodes located over motor areas: FC3, C5, C3, C1, CP3, FC4, C6, C4, C2, and CP4 due to the known local modulation of spindle activity over learning-related brain regions (Cox et al., 2014;Lutz et al., 2021). However, for visualisation purposes (Fig. 3a), the remaining electrodes in the International 10-20 EEG system were also analysed as described below. First, raw data from these channels were down-sampled to 250 Hz (for them to be comparable between the two EEG data acquisition systems) and filtered by Chebyshev Type II infinite impulse response (IIR) filter (passband: f = [0.3–35] Hz; stopband: f < 0.1 Hz & f > 45 Hz). All channels were visually inspected, and the noisy ones were interpolated via triangulation of their nearest neighbours. As a final pre-processing step, we re-referenced the data from CPz to the mastoids (TP9, TP10). A spindle-detection algorithm (Navarrete et al., 2020) was then employed to automatically identify sleep spindles (11–16 Hz). Briefly, the data were filtered in a sigma band by the IIR filter (passband: f = [11–16] Hz; stopband: f < 9 Hz & f > 18 Hz) and the root mean squared (RMS) of the signal was computed using a 300 ms time window. Any event that surpassed the 86.64 percentile (1.5 SD, Gaussian distribution) of the RMS signal was considered a candidate spindle. To fit the spindle detection criteria (Iber, 2007), only the events with unimodal maximum in the 11–16 Hz frequency range in the power spectrum, duration between 0.5 and 2.0 s and at least 5 oscillations, were regarded as sleep spindles (Navarrete et al., 2020).

Any identified spindles that fell (partly or wholly) within a period that had been previously marked as an arousal during sleep scoring were removed. The remaining spindles were separated into those that fell within the cue and no-cue periods. We define the cue period as the 3.5 s time interval after the onset of each tone. Since 3.5 s was the longest inter-trial interval allowed, the cue period essentially covered the time interval from the onset of the first tone in a sequence to 3.5 s after the onset of the last one. In turn, the no-cue period covered the time interval between sequences, that is, from 3.5 to 20.0 s after the onset of the last tone in a sequence. If a spindle fell between the cue and no-cue period, that spindle was removed from further analysis. Thus, only spindles that fell wholly within the cue or no-cue period were included in the analysis.

Spindle density was calculated by dividing the number of spindles at each electrode and in each period of interest (cue period during target sleep stage, no-cue period during target sleep stage) by the duration (in minutes) of that period.

#### 2.7.3 MRI data analysis

MRI data were pre-processed using Statistical Parametric Mapping 12 (SPM12; Wellcome Trust Centre for Neuroimaging, London, UK), running under MATLAB 2018b.

##### 2.7.3.1 fMRI

###### 2.7.3.1.1 Pre-processing

Functional data pre-processing consisted of 1) B0-fieldmap correction using SPM’s fieldmap toolbox (Jezzard & Balaban, 1995); 2) realignment to the mean of the images using a least-squares approach and 6 parameter rigid body spatial transformation to correct for movement artifact (Friston et al., 1995); 3) co-registration with the participants’ individual structural image using rigid body model (Collignon et al., 1995); 4) spatial normalisation to Montreal Neurological Institute brain (MNI space) via the segmentation routine and resampling to 2 mm voxels with a 4th-degree B-spline interpolation (Ashburner & Friston, 2005); and 5) smoothing with 8 mm full-width half maximum (FWHM) Gaussian kernel in line with the literature (Cousins et al., 2016). All steps were performed as implemented in SPM12. B0-fieldmap correction step was omitted for one participant (n = 1) due to technical issues during B0-fieldmap acquisition. No scans had to be excluded due to excessive movement (average translations < 3.3 mm, average rotations < 0.03°).

###### 2.7.3.1.2 Single subject level analysis

Subject-level analysis of the fMRI data was performed using a general linear model (GLM) (Friston et al., 1994), constructed separately for each participant and session. Each block type (cued sequence, uncued sequence, cued random, uncued random) as well as the breaks between the blocks were modelled as five separate, boxcar regressors; button presses were modelled as single events with zero duration. All of these were temporally convolved with a canonical hemodynamic response function (HRF) model embedded in SPM, with no derivatives. To control for movement artifacts, the design matrix also included six head motion parameters, generated during realignment, as non-convolved nuisance regressors. A high-pass filter with a cut-off period of 128 s was implemented in the matrix design to remove low-frequency signal drifts. Finally, serial correlations in the fMRI signal were corrected for using a first-order autoregressive model during restricted maximum likelihood (REML) parameter estimation. Contrast images were obtained for each block type of interest ([cued sequence] and [uncued sequence]), as well as for the difference between the two ([cued > uncued]). The resulting parameter images, generated per participant and per session using a fixed-effects model, were then used as an input for the group-level (i.e., random effects) analysis. Contrast images for the difference between sequence and random blocks were not generated due to the unequal number of each block type performed in the scanner (2 random blocks vs. 24 sequence blocks, per session). This, however, was in accordance with the literature (Cousins et al., 2016).

##### 2.7.3.2 VBM

###### 2.7.3.2.1 Pre-processing

Pre-processing of T1w images was performed in keeping with (Ashburner, 2010) recommendations. Images were first segmented into three tissue probability maps (grey matter, GM; white matter, WM; cerebrospinal fluid, CSF), with two Gaussians used to model each tissue class, very light bias regularisation (0.0001), 60 mm bias FWHM cut-off, and default warping parameters (Ashburner & Friston, 2005). Spatial normalisation was performed with DARTEL (Ashburner, 2007), where the GM and WM segments were used to create customised tissue-class templates and to calculate flow fields. These were subsequently applied to the native GM and WM images of each subject to generate spatially normalised and Jacobian scaled (i.e., modulated) images in the MNI space, resampled at 1.5 mm isotropic voxels. The modulated images were smoothed with an 8 mm FWHM Gaussian kernel, in line with the fMRI analysis. To account for any confounding effects of brain size, we estimated the total intracranial volume (ICV) for each participant at each time point by summing up the volumes of the GM, WM, and CSF probability maps, obtained through segmentation of the original images (Friston et al., 1994). The GM and WM images were then proportionally scaled to the ICV values by means of dividing intensities in each image by the image’s global (i.e., ICV) value before statistical comparisons.

#### 2.7.4 Statistical analysis

All tests conducted were two-tailed, with the significance threshold set at 0.05. For behavioural and EEG data analyses, normality assumption was checked using Shapiro-Wilk test. To compare two related samples, we used paired-samples t-test or Wilcoxon signed-rank test, depending on the Shapiro-Wilk test result. Results are presented as mean ± standard error of the mean (SEM), unless otherwise stated.

##### 2.7.4.1 Behavioural data

Statistical analysis of the behavioural data was performed in R (R Core Team, 2018) or SPSS Statistics 25 (IBM Corp., Armonk, NY, USA) as before (Rakowska et al., 2021). Each dataset (LH, RH, BH) was analysed separately.

To assess the relationship between TMR, SeqSpecS, and Session, we used linear mixed-effects analysis performed on S2–S4, using lme4 package ( Bates et al., 2014 ) in R. We chose linear mixed-effects analysis instead of an ANOVA to avoid listwise deletion due to missing data at S3 and S4 and to account for the non-independence of multiple responses collected over time, in line with previous literature ( Miyamoto et al., 2021 ; Schapiro et al., 2018 ). TMR and Session were entered into the model as categorical (factor) fixed effects without interaction and random intercept was specified for each subject. The final models fitted to the BH, LH, and RH datasets were as follows:

*> model = lmer(early*SeqSpecS*~ Session + TMR + (1|Participant), data = dataset)*
*> model = lmer(late*SeqSpecS*~ Session + TMR + (1|Participant), data = dataset)*

To test for the effect of hand, LH and RH datasets were combined and “hand” (factor) was added as an additional fixed effect:

*> model = lmer(early*SeqSpecS*~ Session + TMR + Hand + (1|Participant), data = dataset)*
*> model = lmer(late*SeqSpecS*~ Session + TMR + Hand + (1|Participant), data = dataset)*

Finally, to explore how the TMR effect evolves from S2 to S4, we entered cueing benefit (calculated using the late SeqSpecS data given no TMR effect on the early SeqSpecS) as the dependent variable and the number of days post-TMR (“time,” integer) as a fixed effect in the following model:

*> model = lmer(CueingBenefit ~ Time + (1|Participant), data = dataset)*

To test for the effect of hand, LH and RH datasets were combined as before:

*> model = lmer(CueingBenefit ~ Time + Hand + (1|Participant), data = dataset)*

Likelihood ratio tests comparing the full model against the model without the effect of interest were performed using the ANOVA function in R to obtain p-values. Post-hoc pairwise comparisons were conducted using the*emmeans*package (Lenth et al., 2019) in R and corrected for multiple comparisons with Holm’s method. Effect sizes were calculated with the*emmeans*package as well.

##### 2.7.4.2 EEG data

Statistical analysis of the EEG data was performed in R (R Core Team, 2018) or SPSS Statistics 25 (IBM Corp., Armonk, NY, USA). Each stimulation period (cue vs. no-cue) and sleep stage (N2, N3, N2 and N3 combined) was analysed separately.

Correlations between our behavioural measures and EEG results were assessed with Pearson’s correlation or Spearman’s Rho (depending on the Shapiro-Wilk test result), using*cor.test*function in the R environment. Any datapoint that was both 1) more than 1.5 IQRs below the first quartile or 1.5 IQRs above the third quartile, and 2) deemed an outlier through visual inspection, was removed from the dataset prior to correlational analysis. False discovery rate (FDR) correction was used to correct for multiple correlations (q < 0.05) (Benjamini & Hochberg, 1995). FDR corrections were based on 3 correlations, given the 3 experimental sessions of interest (S2, S3, S4).

##### 2.7.4.3 MRI data

Group-level analysis of the MRI data was performed either in a Multivariate and Repeated Measures (MRM) toolbox (https://github.com/martynmcfarquhar/MRM) or in SPM12, both running under MATLAB 2018b. All contrasts performed in SPM are outlined inTable S11. All tests conducted were two-tailed, testing for both positive and negative effects. Results were voxel-level corrected for multiple comparisons by family wise error (FWE) correction for the whole brain and for the pre-defined anatomical regions of interest (ROI), with the significance threshold set at p_FWE_< 0.05. For the analysis performed in MRM, p-values were derived from 1,000 permutations, with Wilk’s lambda specified as the test statistic. Pre-defined ROI included 1) bilateral precuneus, 2) bilateral hippocampus and parahippocampus, 3) bilateral dorsal striatum (putamen and caudate), and 4) bilateral sensorimotor cortex (precentral and postcentral gyri). All ROI were selected based on their known involvement in sleep-dependent procedural memory consolidation (Albouy et al., 2013;Debas et al., 2010;Fischer et al., 2005;Walker et al., 2005) and memory reactivation (Brodt et al., 2018;Cousins et al., 2016;Maquet et al., 2000;Rasch et al., 2007;van Dongen et al., 2012). A mask for each ROI was created using an Automated Anatomical Labeling (AAL) atlas in the Wake Forest University (WFU) PickAtlas toolbox (Maldjian et al., 2003). Anatomical localisation of the significant clusters was determined with the automatic labelling of MRIcroGL (https://www.nitrc.org/projects/mricrogl/) based on the AAL atlas. All significant clusters are reported in the tables, but only those with an extent equal to or above 5 voxels are discussed in text and presented in figures.

To account for multiple small volume corrections, any contrast that yielded significant results for either one of our pre-defined ROIs was entered into a voxel-wise permutation analysis with FWE correction within a single mask combining all the pre-defined ROIs. The analysis was performed in MRM with p-values derived from 1,000 permutations and Wilk’s lambda specified as the test statistic.

###### 2.7.4.3.1 fMRI data

To test the effect of TMR on the post-stimulation sessions (S2, S3), one-dimensional contrast images for the [cued] and [uncued] blocks of each session were entered into a repeated-measures TMR-by-Session ANOVA performed in the MRM toolbox.

To compare functional brain activity during the cued and uncued sequence, we carried out one-way t-tests on the [cued > uncued] contrast for S2 (n = 28) and S3 (n = 24) in SPM12. To determine the relationship between the TMR-related functional activity and other factors, we included the behavioural cueing benefit at different time points (S2, S3, S4) as covariates in separate comparisons (Table S11A).

###### 2.7.4.3.2 VBM data

Because structural changes take time to occur, we chose to look for VBM changes between baseline and day 10 (S1 and S3), rather than looking at shorter term effects in S2. Group-level analysis of the structural images was performed separately for GM and WM. First, the pre-processed and proportionally scaled images from S1 and S3 were subtracted from one another (n = 24). To determine the relationship between the long-term structural brain changes and behavioural benefits of TMR, one-sample t-tests were computed in SPM12, with covariates of interest added one at a time. The covariates of interest were the behavioural cueing benefit at S3 and S4. Sex was always specified as a covariate of no interest (nuisance covariate) to control for differences between males and females. Finally, the SPM12 tissue probability maps of GM and WM were thresholded at 50% probability and the resulting binary masks were used in the analyses of the relevant tissue (Ceccarelli et al., 2012).

#### 2.7.5 Results presentation

Plots displaying behavioural results, pairwise comparisons, and relationships between two variables were generated using*ggplot2*(version 3.3.0) (Wickham, 2009) in R.Figure 3awas generated using*ft_topoplotER*function in FieldTrip Toolbox (Buysse et al., 1989).Figure 1was created in Microsoft PowerPoint v16.53. MRI results are presented using MRIcroGL, displayed on the MNI152 standard brain (University of South Carolina, Columbia, SC), exceptFigures S2 and S3which were generated by SPM12 (Wellcome Trust Centre for Neuroimaging, London, UK).

## 3 Results

### 3.1 Srtt

#### 3.1.1 Reaction time and sequence specific skill

**Fig. 2.**
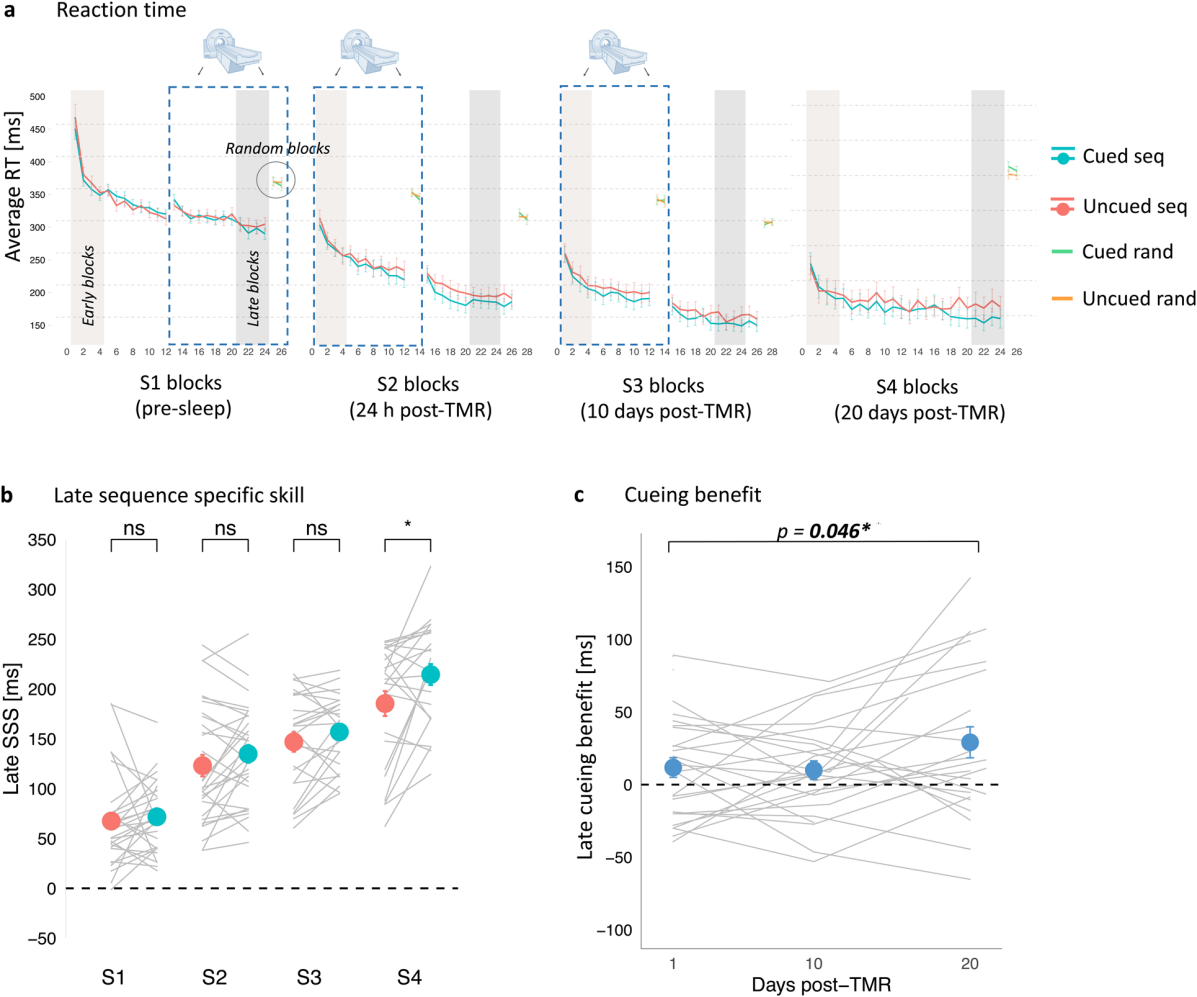
Behavioural benefit of cueing emerges 20 days after the stimulation night. (a) Mean reaction time for the cued sequence (blue), uncued sequence (red), and random blocks (green and orange) of the SRTT performed before sleep (S1), 24 h post-TMR (S2), 10 days post-TMR (S3), and 20 days post-TMR (S4). Error bars depict SEM. Blue dashed rectangle frames mark the SRTT blocks performed during fMRI acquisition. For summary statistics seeTable S1. (b) Mean late SeqSpecS for the cued (blue dots) and uncued (red dots) sequence plotted against experimental sessions (S1–S4). Error bars depict SEM. Grey lines represent individual participants. For statistical analysis results see Tables S2–S4. (c) Mean late SeqSpecS on the uncued sequence subtracted from the cued sequence and plotted over time (number of days post-TMR). The effect of time was significant (seeTable S5). Blue dots represent mean ± SEM calculated for S2, S3, and S4. Grey lines represent cueing benefit for each subject. For (a–c): n = 30 for S1–S2, n = 25 for S3, n = 24 for S4. S1–S4: Session 1–Session 4; RT: reaction time; SeqSpecS: Sequence Specific Skill. *p < 0.05; ns: non-significant. For the effects of TMR and session on each hand seeFigure S1.

Analysis of baseline SRTT performance indicated that participants learned both sequences before sleep and confirmed that any post-sleep differences between the sequences can be regarded as the effect of TMR (see Supplementary Notes: Baseline SRTT Performance andTable S1).Figure 2ashows the mean reaction time (± SEM) for all trials of each SRTT block over the whole length of the study.

Post-sleep SRTT re-test sessions occurred 24.67 h (SD: 0.70) (S2), 10.48 days (SD: 0.92) (S3), and 20.08 days (SD: 0.97) (S4) after session 1 (S1). In line with the methods described in (Cousins et al., 2014,^2016^), SRTT performance was measured by subtracting the mean reaction time on the last or first four blocks of each sequence from that of the random blocks, thereby providing a measure of sequence specific skill for both early and late timepoints. We can then compare these measures to calculate effects of TMR on both early performance (e.g., SRTT performed immediately post-sleep without further practice, thus not requiring post-manipulation practice) and late performance (SRTT measured at the end of post-manipulation practice session, thus including effect of TMR which unfold across subsequent practice), which we refer to as early and late sequence specific skill (SeqSpecS), respectively. To test the effect of cueing on the SeqSpecS (either early or late) over time, we fitted a linear mixed effects model to our behavioural dataset, with TMR and session entered as fixed effects, and participant entered as a random effect. Results of all the likelihood ratio tests comparing the full model against the model without the fixed effect of interest are shown inTable S2.

The linear mixed-effect analysis revealed a main effect of session on both early (X^2^(2) = 175.77, p < 0.001; Table S2Ai) and late SeqSpecS (X^2^(2) = 93.04, p < 0.001; Table S2Aii). Post-hoc comparisons showed a difference between subsequent sessions (S2 vs. S3, S3 vs. S4) (p_adj_< 0.002;Table S3A), suggesting continuous learning over time. All p_adj_values are Holm-corrected.

Inclusion of TMR as a fixed effect improved model fit across all post-stimulation sessions (S2–S4) for late SeqSpecS (X^2^(1) = 11.01, p = 0.001; Table S2Aii), but not early SeqSpecS (X^2^(1) = 1.55, p = 0.214; Table S2Ai). Thus, the linear mixed-effects analysis points to a main effect of TMR on the late SeqSpecS across all post-stimulation sessions. Next, we performed post-hoc comparisons to reveal the session(s) during which late SeqSpecS differed between the two sequences. We found a significant difference between the cued and uncued sequence performance at S4 (20 days post-stimulation, p_adj_= 0.004) but not at S2 (24 h post-stimulation, p_adj_= 0.282) or S3 (10 days post-stimulation, p_adj_= 0.282) (Table S4A;Fig. 2b). Together, these findings point to a main effect of TMR across all post-stimulation sessions, with the difference between the cued and uncued sequence strongest 20 days post-TMR.

Our previous findings on the same task suggest differential consolidation processes for the two hands (Koopman et al., 2020;Rakowska et al., 2021). Thus, we also sought to unpack the effects of TMR and session on each hand separately (see Supplementary Notes: Individual Hands Performance). Although our results suggest greater benefits of TMR on the dominant hand performance at S4 (Fig. S1;Table S2B, C), we found no interaction between hand and TMR (Table S2D). This suggests no difference in how TMR affects the dominant and non-dominant hand consolidation and thus any further analyses testing the relationship between behavioural effects of TMR and other factors involve the both hands dataset only.

#### 3.1.2 Cueing benefit across time

To explore how the TMR effect evolves over time, we used late SeqSpecS, as in prior studies (Cousins et al., 2014;Rakowska et al., 2021). Specifically, we calculated the difference between late SeqSpecS of the cued and uncued sequence for each session, and refer to this as the (late) cueing benefit. Next, we used a linear mixed-effects analysis to determine if cueing benefit changes across post-stimulation time. Inclusion of the number of days post-TMR as the fixed effect improved model fit on the extent of cueing benefit (X^2^(2) = 3.97, p = 0.046;Fig. 2c;Table S5A), suggesting that the effects of TMR may develop in a gradual time-dependent manner.

### 3.2 Correlations with sleep stages

To determine the relationship between sleep parameters derived from sleep stage scoring (Table S6) and the behavioural effect of our manipulation, we correlated the percentage of time spent in stage 2 (N2) and stage 3 (N3) of NREM sleep (the two target stages for our stimulation) with the cueing benefit at each session (S2, S3, S4). Results are presented inTable S7, with no correlation surviving FDR correction (p_adj_> 0.05).

### 3.3 Sleep spindles

**Fig. 3.**
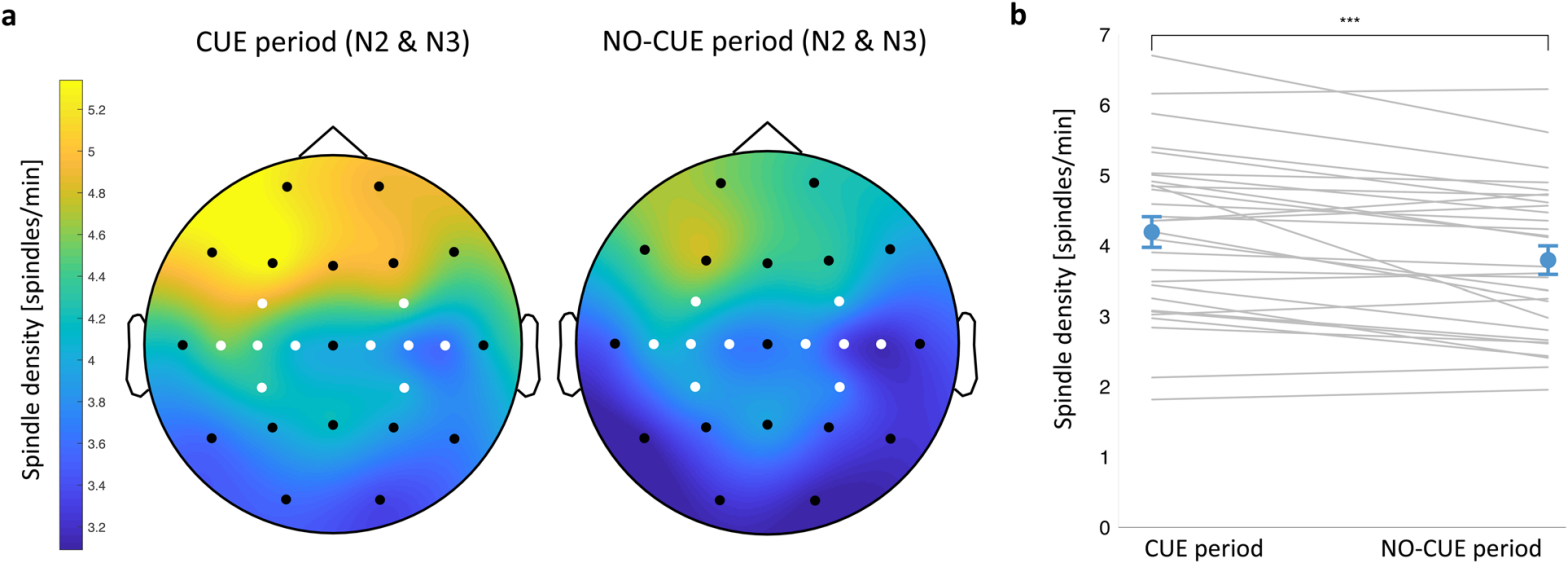
Spindle density increases immediately upon cue onset. (a) Topographic distribution of spindle density (spindles per min) in the cue (left) and no-cue (right) period of NREM sleep (N2 and N3 combined). Motor channels in white. (b) Spindle density averaged over motor channels during the cue period was higher than during the no-cue period. Blue dots represent mean ± SEM. Grey lines represent individual subjects. ***p = 0.001. N2-N3: stage 2–stage 3 of NREM sleep. n = 29. SeeTable S8for summary statistics andTable S9for the relationship between spindle density and cueing benefit.

Given the well-known involvement of sleep spindles in motor sequence memory consolidation (Boutin & Doyon, 2020), we set out to describe electrophysiological changes within the spindle frequency in relation to the cueing procedure. The average spindle density over the task related regions was higher in N2 than in N3 during both the cue period (0–3.5 s after cue onset; t(28) = 4.48, p < 0.001) and the no-cue period (3.5–20 s after the onset of the last cue in the sequence; t(28) = 4.23, p < 0.0001) (paired-samples t-test). Next, we compared spindle density during the cue and the no-cue period for N2 and N3 combined. As in our previous study (Rakowska et al., 2021), we found that the average spindle density during the cue period was higher than during the no-cue period (t(28) = 4.37, p < 0.001; paired-samples t-test;Fig. 3a, b), suggesting that cueing may elicit sleep spindles. The analysis also revealed higher spindle density over the left versus right motor areas for the cue period (t(28) = 2.59, p = 0.015) but not for the no-cue period (t(28) = 1.98, p = 0.057) (paired-samples t-test). Spindle density and the number of spindle events during each period and sleep stage are summarised inTable S8.

Spindle-related changes over brain regions involved in learning (Cox et al., 2014) often predict behavioural performance (Barakat et al., 2013). However, we found no correlation between spindle density averaged over bilateral motor regions and cueing benefit (p_adj_> 0.05;Table S9).

### 3.4 TMR-related changes in fMRI response

**Fig. 4.**
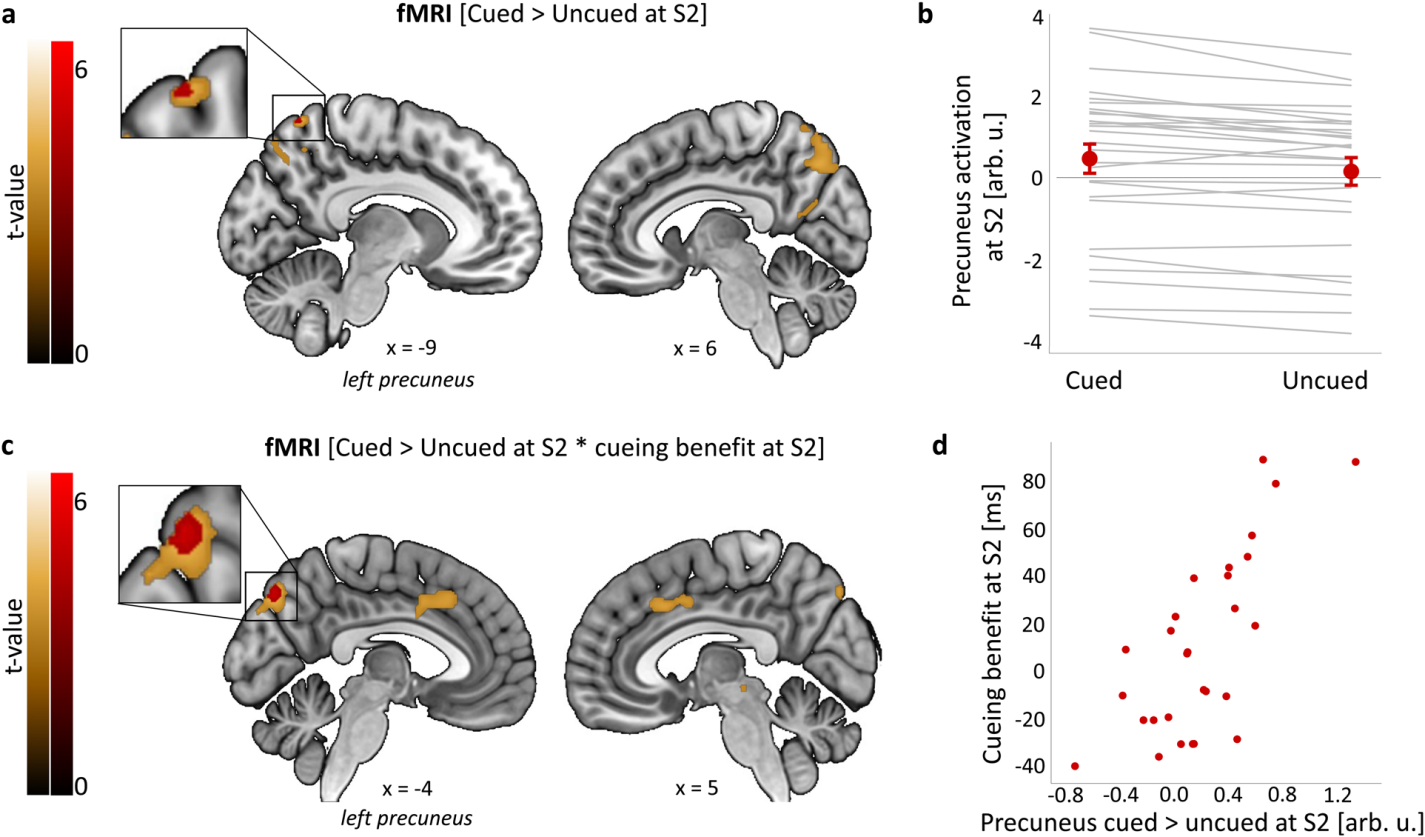
TMR-related functional activity in precuneus. (a, b) TMR-dependent increase in left precuneus activity 24 h post-stimulation. (c, d) Activity for the [cued > uncued] contrast in left precuneus at S2 is positively associated with behavioural cueing benefit at the same time point. (a, c) Group level analysis. In red, colour-coded t-values for each contrast thresholded at a significance level of p_FWE_< 0.05, corrected for multiple voxel-wise comparisons within a pre-defined ROI for bilateral precuneus (Table S10) (for voxel-wise correction within all four ROIs seeTable S13A, B). In gold, colour-coded t-values for each contrast thresholded at a significance level of p < 0.001, uncorrected and without masking. Results are overlaid on a Montreal Neurological Institute (MNI) brain. Note that although the clusters significant at p_FWE_< 0.05 in (a) and (c) fall within the Automated Anatomical Labeling (AAL) definition of precuneus, they do not overlap and their peak coordinates are different (seeTable S10, Bi, Ci, Di). (b, d) Mean functional activity extracted from clusters significant at p_FWE_< 0.05 shown in (a, c). The scatterplots are presented for visualisation purpose only and should not be used for statistical inference. (b) Red dots represent group mean ± SEM. Grey lines represent individual subjects. (d) Each data point represents a single participant. arb. u.: arbitrary units; S2–4: Session 2–4; n = 28 for (a–d). For glass brain fMRI results seeFigure S2.

To test our hypothesis that memory cueing during sleep would engender learning-related changes within precuneus, we performed a TMR-by-Session ANOVA on the fMRI data acquired during sequence performance at S2 (24 h post-TMR) and S3 (10 days post-TMR). In line with our hypothesis, the analysis revealed increased activity in the precuneus (right precuneus, 8, -72, 58) for the main effect of TMR (cued vs. uncued sequence across both S2 and S3) (peak F = 22.67, p = 0.032;Table S10A), but no effect of session or interaction (p > 0.05 ROI corrected). Because we have previously shown cueing-related functional activity the morning after TMR (Cousins et al., 2016) and because both microstructural plasticity and functional engagement of posterior parietal cortex (PPC) have been detected relatively soon after learning (Brodt et al., 2018), we expected to find functional activity changes already at S2. Indeed, a one-way t-test on the [cued > uncued] contrast revealed increased activity in the dorsal-anterior subregion of left precuneus (-9, -62, 66) just 24 h post-TMR (peak T = 4.79, p = 0.020;Fig. 4a, b;Table S10B;Fig. S2A), but no difference between cued and uncued activity at S3 (p > 0.05). These results show that TMR alters functional activity in precuneus, with the TMR-related increase in functional response apparent relatively quickly (i.e., 24 h) post-stimulation.

**Fig. 5.**
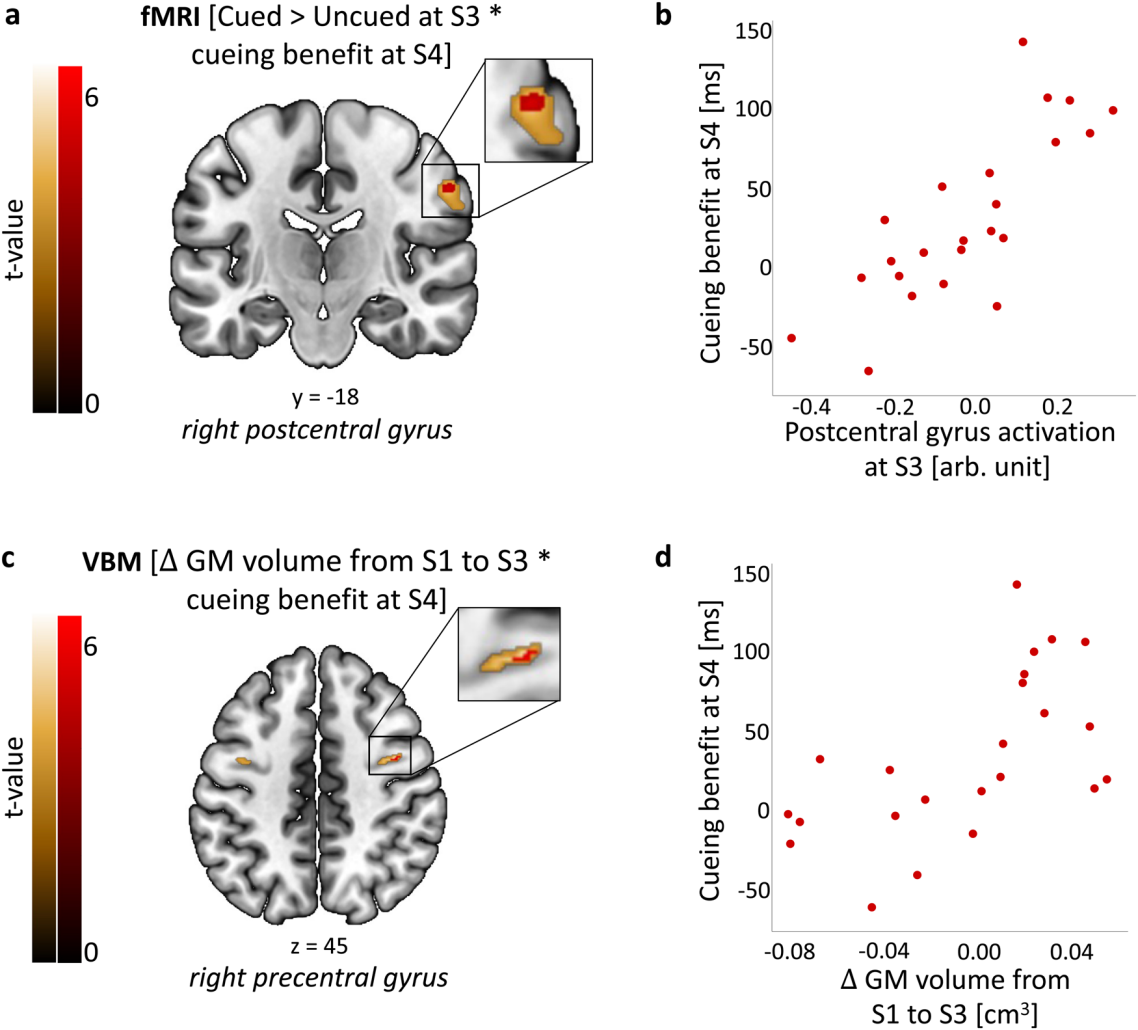
Functional activity and structural brain changes are associated with long-term cueing benefit. (a, b) Activity for the cued > uncued contrast in the right postcentral gyrus at S3 is positively associated with behavioural cueing benefit at S4. (c, d) Grey matter volume in the right precentral gyrus at S3 relative to S1 is positively associated with behavioural cueing benefit at S4. (a, c) Group level analysis. In red, colour-coded t-values for increased fMRI activity (a) and grey matter volume (c), both thresholded at a significance level of p_FWE_< 0.05, corrected for multiple voxel-wise comparisons within a pre-defined ROI for bilateral sensorimotor cortex (Table S12) (for voxel-wise correction within all four ROIs seeTable S13C, D). In gold, colour-coded t-values for increased fMRI activity (a) and grey matter volume (c), both thresholded at a significance level of p < 0.001, uncorrected and without masking. Results are overlaid on a Montreal Neurological Institute (MNI) brain. Colour bars indicate t-values. (b, d) Mean functional activity (b) and grey matter volume (d) extracted from clusters significant at p_FWE_< 0.05 shown in (a, c). The scatterplots are presented for visualisation purpose only and should not be used for statistical inference. Each data point represents a single participant. arb. u.: arbitrary units; GM: grey matter; S1–4: Session 1–4; n = 23. For glass brain fMRI and VBM results seeFigures S2 and S3, respectively.

Next, following the lead of prior authors (Albouy et al., 2013;Debas et al., 2010;Shanahan et al., 2018), we looked for a relationship between post-sleep performance improvements and brain activity differences between the cued and uncued conditions. First, we correlated fMRI responses to the cued > uncued contrast at each post-manipulation session with behavioural regressors collected in that same session. At S2, this revealed that TMR-related functional increase in left dorsal-posterior precuneus was significantly correlated with behavioural cueing benefit, (-4, -78, 46; peak T = 5.18, p = 0.009;Fig. 4c, d; Table S10Ci;Fig. S2B), a finding which survived correction for multiple ROIs (Table S13). Next, to determine how functional responses may predict future behavioural improvements, we correlated the cued > uncued response at each post-manipulation session with behavioural responses from future sessions. This revealed that TMR related responses in the postcentral gyrus at S3 were positively predicting behavioural cueing benefit at S4, around 10 days later (58, -18, 38; peak T = 5.50, p = 0.022;Fig. 5a, b; Tables S10Di and S13;Fig. S2C). Taken together, these two results suggest that activity in dorsal precuneus 24 h post-encoding predicts behavioural effects of cueing in the short-term, while TMR impacts on activation of primary somatosensory cortex 10 days post-encoding may underpin long-term behavioural effects of such cueing.

Further, both results survived correction for the multiple ROIs we examined, although the size of the latter did not exceed 5 voxels and therefore this result should be treated with caution. No significant clusters exceeding 5 voxels were apparent in any of the other ROIs, nor was there any other significant relationship between functional changes and behavioural cueing benefit (Tables S10 and S11).

### 3.5 TMR-related structural plasticity

To determine whether the behavioural effects of TMR were associated with volumetric changes, we performed voxel-based morphometry (VBM) analysis of the T1w scans while taking such changes into account as covariates. Structural changes take time to develop (Draganski et al., 2004;Sagi et al., 2012), and because TMR manipulation was performed within, rather than between, participants we could not use the cued versus uncued comparison when examining brain structure. We therefore examined the relationship between TMR benefits and long-term structural plasticity. Examining changes from S1 to S2 and S2 to S3 in addition to this would have increased the number of comparisons unnecessarily. We first determined the difference between baseline grey and white matter images and equivalent images from the final MRI session collected ~10 days later, (S1 > S3), and conducted a series of analyses in which the behavioural cueing benefit at each post-sleep session was regressed against this (Table S11B). Since we were unsure about the direction of the change, we conducted a two-tailed t-test. This revealed a positive correlation between grey matter (GM) volume change in the right precentral gyrus and cueing benefit at S4 (42, -2, 45; peak T = 6.21, p = 0.020;Fig. 5c, d;Table S12A;Fig. S3A), which survived voxel-wise correction for multiple ROIs (Table S13C). This finding suggests that the TMR related change in GM volume within a sensorimotor structure can predict the long-term behavioural effects of cueing. No correlation with volumetric changes was revealed in either white matter or within other ROIs, and there was no correlation with behavioural cueing benefit at S3, nor when examining shorter-term effects.

## 4 Discussion

In this study, we aimed to determine if repeated reactivation of a memory trace during sleep engenders learning-related changes within the PPC and sensorimotor areas. To this end, we tested the temporal dynamics of the TMR-related changes across structural, functional, electrophysiological, and behavioural measures. Firstly, we showed a main effect of TMR on the SRTT performance across all post-stimulation sessions, with the biggest difference between cued and uncued sequences emerging 20 days post-stimulation. In line with our hypothesis, dorsal precuneus showed a functional response that was related to the manipulation and predicted its behavioural effects the next day. However, over time, this was replaced by an increase in functional activity and volumetric grey matter in somatosensory and motor regions which predicted the longer-term behavioural benefit of our manipulation.

### 4.1 TMR benefits SRTT memories up to 20 days post-manipulation

The strongest behavioural difference between our cued and uncued sequences occurred 20 days post-manipulation, suggesting that the benefits of cueing may last longer than previously believed. This is especially interesting given that neither object-location (Shanahan et al., 2018) nor emotional memory (Groch et al., 2017) seems to benefit from the manipulation even a week later. One-week-later effects of TMR have been reported for implicit biases (Hu et al., 2015), but this failed to replicate (Humiston & Wamsley, 2019). Our prior work showed behavioural effects of TMR 10 days post-manipulation but not 6 weeks later (Rakowska et al., 2021). Hence, the effect of TMR 20 days post-stimulation that we observe here appears to be the longest-term effect reported in the literature so far. This finding suggests that the TMR manipulation starts a process which then unfolds over several weeks, gradually leading to the emergence of behavioural benefits over time. Nevertheless, given the limited availability of independent evidence corroborating the observed pattern in long-term TMR studies, we caution against making broad generalizations based on these findings.

### 4.2 Cueing alters precuneus activation

Dorsal precuneus showed a TMR-dependent (cued > uncued) BOLD increase 24 h post-stimulation. Importantly, this functional response predicted the extent to which TMR impacted on behavioural performance at that same time point, suggesting that repeated reactivation of memory traces during sleep may increase activity in parts of the PPC (such as precuneus) in a behaviourally relevant manner, although the cueing benefit was not yet significant at 24 h. Given that dorsal precuneus is specialised for somato-motor and visual-spatial processing (Zhang & Li, 2012), this finding raises the possibility that visuomotor integration of the reactivated memories may underpin short-term cueing benefits, even if this is not enough to drive the behavioural plasticity. However, the plausibility of such a scenario remains uncertain, emphasizing the need to exercise caution when interpreting our results. Furthermore, PPC has been identified as a hippocampus-independent memory store, whereby both hippocampal activity and connectivity with PPC decrease soon after encoding, but (conversely) PPC activity increases over the next 24 h, as an independent memory representation builds up (Brodt et al., 2016). We believe that sleep plays a crucial role in this process and that the reactivation-mediated reorganisation of memories between the hippocampal-dependent short-term store and neocortex-dependent long-term store (Born et al., 2006;Diekelmann & Born, 2010) fosters engram development in the precuneus. We speculate that memory reactivation could be taking place in precuneus (Himmer et al., 2021), such that SRTT memories are stored and processed in the same location. Indeed, precuneus has repeatedly been implicated in memory formation, retrieval, and storage (Gilmore et al., 2015;Myskiw & Izquierdo, 2012;Wagner et al., 2005). Multiple studies have also linked precuneus with episodic memory, both when imagery is required (Buckner et al., 1995;Fletcher et al., 1996;Halsband et al., 1998;Henson et al., 1999) and when it is not (Krause et al., 1999;Platel et al., 2003;Schmidt et al., 2002), seeCavanna and Trimble (2006)for a review. This structure is traditionally associated with the motor system (Cohen & Andersen, 2002;Shadmehr & Holcomb, 1997), with several studies showing a role for precuneus in finger tapping (Hanakawa et al., 2003) and bimanual motor tasks (Fattinger et al., 2017;Wenderoth et al., 2005). Further, precuneus has also recently been implicated in declarative memory processing (Brodt et al., 2016,^2018^). This is particularly relevant here, as the SRTT is not purely procedural, but is thought to have a declarative component (Albouy et al., 2013,^2008^). Our results build on all of this to suggest that precuneus may be involved in early (across 24 h) consolidation of memories that are reactivated during sleep.

Although we showed that TMR-related functional activity in precuneus is associated with behavioural cueing benefit 24 h post-manipulation, it is important to note that cueing benefit was not significant at this time point when considered in isolation. Our prior studies of the SRTT have shown a cueing benefit from TMR immediately after the manipulation (Cousins et al., 2014,^2016^;Koopman et al., 2020); however, we have previously argued that jittering of our TMR cues as we did in the current paradigm could detract from this (Rakowska et al., 2021). Thus, randomising the inter-trial-interval between the TMR sounds during sleep could disrupt the temporal dynamics of sequence replay, decreasing the predictability of sequence elements. This may have delayed the impact of this manipulation on behaviour, such that behavioural impacts of TMR were not significant until 20 days post-manipulation. Even so, the absence of a TMR-related behavioural plasticity 10 days after cueing was unexpected given that cueing benefit was apparent at this time point in our prior study using jittered TMR (Rakowska et al., 2021). Interestingly, the only session during which we observed a significant cueing benefit was the one which was performed online and in participants’ own homes using a computer keyboard, so one possibility is that doing the task while lying down in the MRI scanner, and with somewhat clunky MR-safe button boxes, impacted on the behavioural effects of TMR which would otherwise have been apparent. However, a between-session comparison of reaction times argues against this, since it revealed that the participants were faster in the MRI environment (S3) than when performing the task on a PC (S4), with variance equal in the two sessions (Fig. S5). The MRI environment could still have influenced our behavioural results, but there is no reason to expect that it would impact differentially on the two sequences and thus the difference between them (i.e., cueing benefit).

### 4.3 Plasticity within sensorimotor regions predicts long-term cueing benefits

Our data show that both the functional activation and the volumetric grey matter increase in the sensorimotor cortex at 10 days post-TMR predict long-term behavioural cueing benefits. Thus, TMR-related functional activity in the right postcentral gyrus 10 days post-stimulation predicts behavioural benefits 20 days post-stimulation. Furthermore, an increase in grey matter volume in the right precentral gyrus over the first 10 days post-stimulation predicts the same behavioural benefits. The temporal lag between changes in the brain and the delayed changes we observed in behaviour may seem surprising at first glance, but we feel that these results make sense in that they suggest that a slowly evolving reorganisation of sensorimotor representations may underpin consolidation of TMR benefit to the SRTT over a 20-day timescale. It takes time for this change to become sufficiently large to be reflected in a significant behavioural benefit. In fact, the timescale at which this behavioural benefit emerges differs between studies, as it only becomes apparent at day 20 in the current report, while it was already apparent 24 h post-manipulation in our prior examination of this task (Rakowska et al., 2021). We speculate that such differences in the time needed for this effect to unfold are governed by aspects of our design and the circumstances in which participants performed the task (e.g., being in the scanner vs. at home, see our discussion insection 3.2*Cueing Alters Precuneus Activation*), but individual differences in learning strength, sleep patterns, and even brain morphology could also play a role (Abdellahi et al., 2023;Buch et al., 2021;Ebrahimi & Ostry, 2024;Kumar et al., 2019;Rakowska et al., 2022). The somatosensory cortex has been shown to be essential for motor memory consolidation, since disruption of this region after learning dramatically impairs subsequent retention of a motor task. Notably, disruption of the primary motor cortex at the same timepoint has no impact on retention (Ebrahimi & Ostry, 2024;Kumar et al., 2019). The first excitability changes during motor skill learning have been shown to occur in the somatosensory cortex and these predicted extent of subsequent learning, while changes in motor cortex excitability did not (Hanakawa et al., 2003). Furthermore, wakeful replay of motor sequences has been shown to involve somatosensory cortex (Buch et al., 2021). Our results build on this prior literature to suggest that our TMR manipulation leads to both structural and functional changes in the sensorimotor cortex that evolve over time and predict TMR-related performance benefit. However, they should be treated with caution due to the small sample size and the fact that behavioural data at day 20 were collected remotely and showed a large standard deviation.

### 4.4 The role of N2 and sleep spindles

There is a fundamental similarity between the reactivation of memory traces via TMR and repeated encoding-retrieval episodes during wake, both of which have been shown to engender rapid memory engram formation within the precuneus (Antony et al., 2017;Brodt et al., 2018). Indeed, repeated retrieval is a powerful way to consolidate memories and shares a lot of parallels with offline reactivation (Antony et al., 2017). However, in line with other studies (Himmer et al., 2019), we argue that the role of sleep goes beyond simply allowing an opportunity for more rehearsal. Both N2 (Laventure et al., 2016;Nishida & Walker, 2007) and sleep spindles (Boutin & Doyon, 2020) have been consistently implicated in motor sequence memory consolidation. Although we found no relationship between behavioural cueing benefit and either the time spent in N2 or spindle density, we did find a surge in spindle density during the cue period relative to the no-cue period. This is in line with our prior report (Rakowska et al., 2021) and suggests that auditory cueing may elicit sleep spindles. Even though this could also indicate an immediate processing of memory traces (Antony et al., 2018;Cairney et al., 2018), a comparison between the electrophysiological response to cues versus control sounds would be necessary to confirm the relationship between spindles and memory cueing. Such work is unfortunately outside the scope of this report, as we did not apply control sounds.

### 4.5 The search for an engram

A neuronal ensemble that holds a representation of a stable memory is known as an engram (Tonegawa et al., 2018). The term “engram” also refers to the physical brain changes that are induced by learning and that enable memory recall (Josselyn et al., 2015). Due to their widely distributed and dynamic nature, engrams have long remained elusive. However, recent technological advances allow us to study memory engrams in humans (Josselyn et al., 2015). PPC, for instance, has received increasing attention in memory research (Gilmore et al., 2015), and the precuneus is a subregion of PPC that has been shown to undergo learning-dependent plasticity, fulfilling all criteria for a memory engram (Brodt et al., 2018). These defining criteria require an engram to 1) emerge as a result of encoding and reflect the content of the encoded information, 2) engender a persistent, physical change in the underlying substrate that 3) enable memory retrieval, and 4) exist in a dormant or inactive state, that is, between encoding and retrieval processes (Josselyn et al., 2015). Evidence for a relationship between engram formation and memory reactivation during sleep has so far been lacking. While previous literature suggests that changes in the precuneus alone fulfil all proposed criteria for an engram (Brodt et al., 2018), our data show no TMR-related structural changes in this region, and thus fail to fulfil criterion 2. This could be due to our use of different MRI modalities (i.e., structural rather than microstructural MRI as inBrodt et al. (2018)). Nevertheless, if our results are considered collectively across regions, we can argue that they do fulfil the criteria for an engram. Specifically, we observed that TMR-related activity in the precuneus and postcentral gyrus predicted behavioural benefit of TMR at S2 and S4, respectively. These responses could therefore reflect the encoded information (criterion 1), and enable memory recall (criterion 3), and that the precentral gyrus undergoes structural changes (criterion 2) which develop over a relatively long period of time (criterion 4). Taken in this way, our results would suggest that memory reactivation during sleep could support the development and evolution of an engram that encompasses several cortical areas, but we acknowledge this is speculative.

## 5 Conclusion

We show that the behavioural benefits of memory cueing in NREM sleep develop over time and can be significant 20 days post-encoding. Increased TMR-related activity of dorsal precuneus underpins the short-term effects of stimulation (over 24 h), whereas sensorimotor regions support the long-term effects (over 20 days). These results advance our understanding of the neural changes associated with long-term offline skill consolidation. They also shed new light on the TMR-induced processes that unfold over several nights after auditory cueing.

## Supplementary Material

Supplementary Material

## Data Availability

All data collected during the study, scripts that delivered experimental tasks, and codes used to conduct the analyses are publicly available at: DOI 10.17605/OSF.IO/Y43SB. A custom-made interface used to perform sleep scoring can be accessed at:https://github.com/mnavarretem/psgScore.

## References

[b1] Abdellahi , M. E. , Koopman , A. C. , Treder , M. S. , & Lewis , P. A. ( 2023 ). Targeted memory reactivation in human REM sleep elicits detectable reactivation . Elife , 12 , e84324 . 10.7554/eLife.84324 37350572 PMC10425171

[b2] Albouy , G. , Sterpenich , V. , Balteau , E. , Vandewalle , G. , Desseilles , M. , Dang-Vu , T. , Darsaud , A. , Ruby , P. , Luppi , P. , Degueldre , C. , Peigneux , P. , Luxen , A. , & Maquet , P. ( 2008 ). Both the hippocampus and striatum are involved in consolidation of motor sequence memory . Neuron , 58 ( 2 ), 261 – 272 . 10.1016/j.neuron.2008.02.008 18439410

[b3] Albouy , G. , Sterpenich , V. , Vandewalle , G. , Darsaud , A. , Gais , S. , Rauchs , G. , Desseilles , M. , Boly , M. , Dang-Vu , T. , Balteau , E. , Degueldre , C. , Phillips , C. , Luxen , A. , & Maquet , P. ( 2013 ). Interaction between hippocampal and striatal systems predicts subsequent consolidation of motor sequence memory . PLoS One , 8 ( 3 ), e59490 . 10.1371/journal.pone.0059490 23533626 PMC3606142

[b4] Antony , J. W. , Ferreira , C. S. , Norman , K. A. , & Wimber , M. ( 2017 ). Retrieval as a fast route to memory consolidation . Trends in Cognitive Sciences , 21 ( 8 ), 573 – 576 . 10.1016/j.tics.2017.05.001 28583416 PMC5912918

[b5] Antony , J. W. , Gobel , E. W. , O’hare , J. K. , Reber , P. J. , & Paller , K. A. ( 2012 ). Cued memory reactivation during sleep influences skill learning . Nature Neuroscience , 15 ( 8 ), 1114 – 1116 . 10.1038/nn.3152 22751035 PMC3498459

[b6] Antony , J. W. , Piloto , L. , Wang , M. , Pacheco , P. , Norman , K. A. , & Paller , K. A. ( 2018 ). Sleep spindle refractoriness segregates periods of memory reactivation . Current Biology , 28 ( 11 ), 1736 – 1743 . 10.1016/j.cub.2018.04.020 29804809 PMC5992601

[b7] Ashburner , J. ( 2007 ). A fast diffeomorphic image registration algorithm . NeuroImage , 38 ( 1 ), 95 – 113 . 10.1016/j.neuroimage.2007.07.007 17761438

[b8] Ashburner , J. ( 2010 ). VBM tutorial. Wellcome Trust Centre for Neuroimaging . http://www.fil.ion.ucl.ac.uk/~john/misc/VBMclass10.pdf

[b9] Ashburner , J. , & Friston , K. J. ( 2005 ). Unified segmentation . NeuroImage , 26 ( 3 ), 839 – 851 . 10.1016/j.neuroimage.2005.02.018 15955494

[b10] Barakat , M. , Carrier , J. , Debas , K. , Lungu , O. , Fogel , S. , Vandewalle , G. , Hoge , R., D. , Bellec , P. , Karni , A. , Ungerleider , L. , G., Benali , H. , & Doyon , J. ( 2013 ). Sleep spindles predict neural and behavioral changes in motor sequence consolidation . Human Brain Mapping , 34 ( 11 ), 2918 – 2928 . 10.1002/hbm.22116 22674673 PMC6870513

[b11] Bates , D. , Mächler , M. , Bolker , B. , & Walker , S. ( 2014 ). Fitting linear mixed-effects models using lme4 . arXiv . 10.48550/arXiv.1406.5823

[b12] Bendor , D. , & Wilson , M. A. ( 2012 ). Biasing the content of hippocampal replay during sleep . Nature Neuroscience , 15 ( 10 ), 1439 – 1444 . 10.1038/nn.3203 22941111 PMC4354843

[b13] Benjamini , Y. , & Hochberg , Y. ( 1995 ). Controlling the false discovery rate: A practical and powerful approach to multiple testing . Journal of the Royal Statistical Society: Series B (Methodological) , 57 ( 1 ), 289 – 300 . 10.1111/j.2517-6161.1995.tb02031.x

[b14] Berry , R. B. , Gamaldo , C. E. , Harding , S. M. , Brooks , R. , Lloyd , R. M. , Vaughn , B. V. , & Marcus , C. L. ( 2015 ). AASM scoring manual version 2.2 updates: New chapters for scoring infant sleep staging and home sleep apnea testing . Journal of Clinical Sleep Medicine , 11 ( 11 ), 1253 – 1254 . 10.5664/jcsm.5176 26446251 PMC4623121

[b15] Born , J. , Rasch , B. , & Gais , S. ( 2006 ). Sleep to remember . The Neuroscientist , 12 ( 5 ), 410 – 424 . 10.1177/1073858406292647 16957003

[b16] Born , J. , & Wilhelm , I. ( 2012 ). System consolidation of memory during sleep . Psychological Research , 76 , 192 – 203 . 10.1007/s00426-011-0335-6 21541757 PMC3278619

[b17] Boutin , A. , & Doyon , J. ( 2020 ). A sleep spindle framework for motor memory consolidation . Philosophical Transactions of the Royal Society B , 375 ( 1799 ), 20190232 . 10.1098/rstb.2019.0232 PMC7209914 32248783

[b18] Brainard , D. H. , & Vision , S. ( 1997 ). The psychophysics toolbox . Spatial Vision , 10 ( 4 ), 433 – 436 . http://www.ncbi.nlm.nih.gov/pubmed/9176952 9176952

[b19] Brodt , S. , Gais , S. , Beck , J. , Erb , M. , Scheffler , K. , & Schönauer , M. ( 2018 ). Fast track to the neocortex: A memory engram in the posterior parietal cortex . Science , 362 ( 6418 ), 1045 – 1048 . 10.1126/science.aau2528 30498125

[b20] Brodt , S. , Pöhlchen , D. , Flanagin , V. L. , Glasauer , S. , Gais , S. , & Schönauer , M. ( 2016 ). Rapid and independent memory formation in the parietal cortex . Proceedings of the National Academy of Sciences of the United States of America , 113 ( 46 ), 13251 – 13256 . 10.1073/pnas.1605719113 27803331 PMC5135314

[b21] Buch , E. R. , Claudino , L. , Quentin , R. , Bönstrup , M. , & Cohen , L. G. ( 2021 ). Consolidation of human skill linked to waking hippocampo-neocortical replay . Cell Reports , 35 ( 10 ), 109193 . 10.1016/j.celrep.2021.109193 34107255 PMC8259719

[b22] Buckner , R. L. , Petersen , S. E. , Ojemann , J. G. , Miezin , F. M. , Squire , L. R. , & Raichle , M. E. ( 1995 ). Functional anatomical studies of explicit and implicit memory retrieval tasks . Journal of Neuroscience , 15 ( 1 ), 12 – 29 . 10.1523/jneurosci.15-01-00012.1995 7823123 PMC6578325

[b23] Buysse , D. J. , III Reynolds , F. C. , Monk , T. H. , Berman , S. R. , & Kupfer , D. J. ( 1989 ). The Pittsburgh Sleep Quality Index: A new instrument for psychiatric practice and research . Psychiatry Research , 28 ( 2 ), 193 – 213 . 10.1016/0165-1781(89)90047-4 2748771

[b24] Cairney , S. A. , Marj El , N., & Staresina , B. P. ( 2018 ). Memory consolidation is linked to spindle-mediated information processing during sleep . Current Biology , 28 ( 6 ), 948 – 954 . 10.1016/j.cub.2018.01.087 29526594 PMC5863764

[b25] Cavanna , A. E. , & Trimble , M. R. ( 2006 ). The precuneus: A review of its functional anatomy and behavioural correlates . Brain , 129 ( 3 ), 564 – 583 . 10.1093/brain/awl004 16399806

[b26] Ceccarelli , A. , Jackson , J. S. , Tauhid , S. , Arora , A. , Gorky , J. , Dell’Oglio , E. , Bakshi , A. , Chitnis , T. , Khoury , S., J. , Weiner , H. , L., Guttmann , C. , R., G., Bakshi , R. , & Neema , M. ( 2012 ). The impact of lesion in-painting and registration methods on voxel-based morphometry in detecting regional cerebral gray matter atrophy in multiple sclerosis . American Journal of Neuroradiology , 33 ( 8 ), 1579 – 1585 . 10.3174/ajnr.A3083 22460341 PMC3425668

[b27] Cohen , Y. E. , & Andersen , R. A. ( 2002 ). A common reference frame for movement plans in the posterior parietal cortex . Nature Reviews Neuroscience , 3 ( 7 ), 553 – 562 . 10.1038/nrn873 12094211

[b28] Collignon , A. , Maes , F. , Delaere , D. , Vandermeulen , D. , Suetens , P. , & Marchal , G. ( 1995 ). Automated multi-modality image registration based on information theory . In Information Processing in Medical Imaging , (Vol. 3 , No. 6 , pp. 263 – 274 ).

[b29] Cousins , J. N. , El-Deredy , W. , Parkes , L. M. , Hennies , N. , & Lewis , P. A. ( 2014 ). Cued memory reactivation during slow-wave sleep promotes explicit knowledge of a motor sequence . Journal of Neuroscience , 34 ( 48 ), 15870 – 15876 . 10.1523/jneurosci.1011-14.2014 25429129 PMC4244461

[b30] Cousins , J. N. , El-Deredy , W. , Parkes , L. M. , Hennies , N. , & Lewis , P. A. ( 2016 ). Cued reactivation of motor learning during sleep leads to overnight changes in functional brain activity and connectivity . PLoS Biology , 14 ( 5 ), e1002451 . 10.1371/journal.pbio.1002451 27137944 PMC4854410

[b31] Cox , R. , Hofman , W. F. , de Boer , M. , & Talamini , L. M. ( 2014 ). Local sleep spindle modulations in relation to specific memory cues . NeuroImage , 99 , 103 – 110 . 10.1016/j.neuroimage.2014.05.028 24852461

[b32] Debas , K. , Carrier , J. , Orban , P. , Barakat , M. , Lungu , O. , Vandewalle , G. , Tahar , A., H. , Bellec , P. , Karni , A. , Ungerleider , L. , G., Benali , H. , & Doyon , J. ( 2010 ). Brain plasticity related to the consolidation of motor sequence learning and motor adaptation . Proceedings of the National Academy of Sciences of the United States of America , 107 ( 41 ), 17839 – 17844 . 10.1073/pnas.1013176107 20876115 PMC2955095

[b33] Deuker , L. , Olligs , J. , Fell , J. , Kranz , T. A. , Mormann , F. , Montag , C. , Reuter , M. , Elger , C., E. , & Axmacher , N. ( 2013 ). Memory consolidation by replay of stimulus-specific neural activity . Journal of Neuroscience , 33 ( 49 ), 19373 – 19383 . 10.1523/JNEUROSCI.0414-13.2013 24305832 PMC6618788

[b34] Diekelmann , S. , & Born , J. ( 2010 ). The memory function of sleep . Nature Reviews Neuroscience , 11 ( 2 ), 114 – 126 . 10.1038/nrn2762 20046194

[b35] Draganski , B. , Gaser , C. , Busch , V. , Schuierer , G. , Bogdahn , U. , & May , A. ( 2004 ). Changes in grey matter induced by training . Nature , 427 ( 6972 ), 311 – 312 . 10.1038/427311a 14737157

[b36] Ebrahimi , S. , & Ostry , D. J. ( 2024 ). The human somatosensory cortex contributes to the encoding of newly learned movements . Proceedings of the National Academy of Sciences of the United States of America , 121 ( 6 ), e2316294121 . 10.1073/pnas.2316294121 38285945 PMC10861869

[b37] Fattinger , S. , de Beukelaar , T. T. , Ruddy , K. L. , Volk , C. , Heyse , N. C. , Herbst , J. A. , Hahnloser , R., H. , R., Wenderoth , N. , & Huber , R. ( 2017 ). Deep sleep maintains learning efficiency of the human brain . Nature Communications , 8 ( 1 ), 15405 . 10.1038/ncomms15405 PMC5458149 28530229

[b38] Fischer , S. , Nitschke , M. F. , Melchert , U. H. , Erdmann , C. , & Born , J. ( 2005 ). Motor memory consolidation in sleep shapes more effective neuronal representations . Journal of Neuroscience , 25 ( 49 ), 11248 – 11255 . 10.1523/JNEUROSCI.1743-05.2005 16339020 PMC6725908

[b39] Fletcher , P. C. , Shallice , T. , Frith , C. D. , Frackowiak , R. S. J. , & Dolan , R. J. ( 1996 ). Brain activity during memory retrieval: The influence of imagery and semantic cueing . Brain , 119 ( 5 ), 1587 – 1596 . 10.1093/brain/119.5.1587 8931582

[b40] Friston , K. J. , Frith , C. D. , Frackowiak , R. S. , & Turner , R. ( 1995 ). Characterizing dynamic brain responses with fMRI: A multivariate approach . NeuroImage , 2 ( 2 ), 166 – 172 . 10.1006/nimg.1995.1019 9343599

[b41] Friston , K. J. , Holmes , A. P. , Worsley , K. J. , Poline , J. P. , Frith , C. D. , & Frackowiak , R. S. ( 1994 ). Statistical parametric maps in functional imaging: A general linear approach . Human Brain Mapping , 2 ( 4 ), 189 – 210 . 10.1002/hbm.460020402

[b42] Gilmore , A. W. , Nelson , S. M. , & McDermott , K. B. ( 2015 ). A parietal memory network revealed by multiple MRI methods . Trends in Cognitive Sciences , 19 ( 9 ), 534 – 543 . 10.1016/j.tics.2015.07.004 26254740

[b43] Groch , S. , Preiss , A. , McMakin , D. L. , Rasch , B. , Walitza , S. , Huber , R. , & Wilhelm , I. ( 2017 ). Targeted reactivation during sleep differentially affects negative memories in socially anxious and healthy children and adolescents . Journal of Neuroscience , 37 ( 9 ), 2425 – 2434 . 10.1523/JNEUROSCI.1912-16.2017 28143960 PMC6596843

[b44] Halsband , U. , Krause , B. J. , Schmidt , D. , Herzog , H. , Tellmann , L. , & Müller-Gärtner , H. W. ( 1998 ). Encoding and retrieval in declarative learning: A positron emission tomography study . Behavioural Brain Research , 97 ( 1-2 ), 69 – 78 . 10.1016/S0166-4328(98)00028-X 9867232

[b45] Hanakawa , T. , Immisch , I. , Toma , K. , Dimyan , M. A. , Van Gelderen , P. , & Hallett , M. ( 2003 ). Functional properties of brain areas associated with motor execution and imagery . Journal of Neurophysiology , 89 ( 2 ), 989 – 1002 . 10.1152/jn.00132.2002 12574475

[b46] Henson , R. N. A. , Shallice , T. , & Dolan , R. J. ( 1999 ). Right prefrontal cortex and episodic memory retrieval: A functional MRI test of the monitoring hypothesis . Brain , 122 ( 7 ), 1367 – 1381 . 10.1093/brain/122.7.1367 10388802

[b47] Himmer , L. , Bürger , Z. , Fresz , L. , Maschke , J. , Wagner , L. , Brodt , S. , Braun , C. , & Gais , S. ( 2021 ). Localizing spontaneous memory reprocessing during human sleep . BioRxiv . 10.1101/2021.11.29.470230

[b48] Himmer , L. , Schönauer , M. , Heib , D. P. J. , Schabus , M. , & Gais , S. ( 2019 ). Rehearsal initiates systems memory consolidation, sleep makes it last . Science Advances , 5 ( 4 ), eaav1695 . 10.1126/sciadv.aav1695 31032406 PMC6482015

[b49] Hoddes , E. , Zarcone , V. , Smythe , H. , Phillips , R. , & Dement , W. C. ( 1973 ). Quantification of sleepiness: A new approach . Psychophysiology , 10 ( 4 ), 431 – 436 . 10.1111/j.1469-8986.1973.tb00801.x 4719486

[b50] Hu , X. , Antony , J. W. , Creery , J. D. , Vargas , I. M. , Bodenhausen , G. V. , & Paller , K. A. ( 2015 ). Unlearning implicit social biases during sleep . Science , 348 ( 6238 ), 1013 – 1015 . 10.1126/science.aaa3841 26023137 PMC4467959

[b51] Humiston , G. B. , & Wamsley , E. J. ( 2019 ). Unlearning implicit social biases during sleep: A failure to replicate . PLoS One , 14 ( 1 ), e0211416 . 10.1371/journal.pone.0211416 30682167 PMC6347202

[b52] Iber , C. ( 2007 ). The AASM manual for the scoring of sleep and associated events: Rules, terminology, and technical specification. (No Title) .

[b53] Jezzard , P. , & Balaban , R. S. ( 1995 ). Correction for geometric distortion in echo planar images from B0 field variations . Magnetic Resonance in Medicine , 34 ( 1 ), 65 – 73 . 10.1002/mrm.1910340111 7674900

[b54] Josselyn , S. A. , Köhler , S. , & Frankland , P. W. ( 2015 ). Finding the engram . Nature Reviews Neuroscience , 16 ( 9 ), 521 – 534 . 10.1038/nrn4000 26289572

[b55] Kodama , M. , Ono , T. , Yamashita , F. , Ebata , H. , Liu , M. , Kasuga , S. , & Ushiba , J. ( 2018 ). Structural gray matter changes in the hippocampus and the primary motor cortex on an-hour-to-one-day scale can predict arm-reaching performance improvement . Frontiers in Human Neuroscience , 12 , 209 . 10.3389/fnhum.2018.00209 29988447 PMC6024594

[b56] Koopman , A. C. , Abdellahi , M. E. , Belal , S. , Rakowska , M. , Metcalf , A. , Śledziowska , M. , Hunter , T. & Lewis , P. ( 2020 ). Targeted memory reactivation of a serial reaction time task in SWS, but not REM, preferentially benefits the non-dominant hand . BioRxiv . 10.1101/2020.11.17.381913

[b57] Krause , B. J. , Schmidt , D. , Mottaghy , F. M. , Taylor , J. , Halsband , U. , Herzog , H. , Tellmann , L. , & Müller-Gärtner , H. W. ( 1999 ). Episodic retrieval activates the precuneus irrespective of the imagery content of word pair associates: A PET study . Brain , 122 ( 2 ), 255 – 263 . 10.1093/brain/122.2.255 10071054

[b58] Kumar , N. , Manning , T. F. , & Ostry , D. J. ( 2019 ). Somatosensory cortex participates in the consolidation of human motor memory . PLoS Biology , 17 ( 10 ), e3000469 . 10.1371/journal.pbio.3000469 31613874 PMC6793938

[b59] Laventure , S. , Fogel , S. , Lungu , O. , Albouy , G. , Sévigny-Dupont , P. , Vien , C. , Sayour , C. , Carrier , J. , Benali , H. , & Doyon , J. ( 2016 ). NREM2 and sleep spindles are instrumental to the consolidation of motor sequence memories . PLoS Biology , 14 ( 3 ), e1002429 . 10.1371/journal.pbio.1002429 27032084 PMC4816304

[b60] Lenth , R. , Singmann , H. , Love , J. , Buerkner , P. , & Herve , M. ( 2019 ). Emmeans: Estimated marginal means, aka least-squares means (Version 1.3.4) . Emmeans Estim Marg Means Aka Least-Sq Means . https://cran.r-project.org/package=emmeans

[b61] Loganathan , R. ( 2014 ). The role of sleep in motor learning . PostDoc Journal . 10.14304/SURYA.JPR.V2N4.2

[b62] Lutz , N. D. , Admard , M. , Genzoni , E. , Born , J. , & Rauss , K. ( 2021 ). Occipital sleep spindles predict sequence learning in a visuo-motor task . Sleep , 44 ( 8 ), zsab056 . 10.1093/sleep/zsab056 33743012 PMC8361350

[b63] Maldjian , J. A. , Laurienti , P. J. , Kraft , R. A. , & Burdette , J. H. ( 2003 ). An automated method for neuroanatomic and cytoarchitectonic atlas-based interrogation of fMRI data sets . NeuroImage , 19 ( 3 ), 1233 – 1239 . 10.1016/S1053-8119(03)00169-1 12880848

[b64] Maquet , P. , Laureys , S. , Peigneux , P. , Fuchs , S. , Petiau , C. , Phillips , C. , Aerts , J. , Del Fiore , G. , Degueldre , C. , Meulemans , T. , Luxen , A. , Franck , G. , Van Der Linden , M. , Smith , C. , & Cleeremans , A. ( 2000 ). Experience-dependent changes in cerebral activation during human REM sleep . Nature Neuroscience , 3 ( 8 ), 831 – 836 . 10.1038/77744 10903578

[b65] McClelland , J. L. , McNaughton , B. L. , & O’Reilly , R. C. ( 1995 ). Why there are complementary learning systems in the hippocampus and neocortex: Insights from the successes and failures of connectionist models of learning and memory . Psychological Review , 102 ( 3 ), 419 . 10.1037/0033-295X.102.3.419 7624455

[b66] Miyamoto , D. , Marshall , W. , Tononi , G. , & Cirelli , C. ( 2021 ). Net decrease in spine-surface GluA1-containing AMPA receptors after post-learning sleep in the adult mouse cortex . Nature Communications , 12 ( 1 ), 2881 . 10.1038/s41467-021-23156-2 PMC8129120 34001888

[b67] Myskiw , J. C. , & Izquierdo , I. ( 2012 ). Posterior parietal cortex and long-term memory: Some data from laboratory animals . Frontiers in Integrative Neuroscience , 6 , 8 . 10.3389/fnint.2012.00008 22375107 PMC3287050

[b68] Navarrete , M. , Schneider , J. , Ngo , H. V. V. , Valderrama , M. , Casson , A. J. , & Lewis , P. A. ( 2020 ). Examining the optimal timing for closed-loop auditory stimulation of slow-wave sleep in young and older adults . Sleep , 43 ( 6 ), zsz315 . 10.1093/sleep/zsz315 31872860 PMC7294407

[b69] Nishida , M. , & Walker , M. P. ( 2007 ). Daytime naps, motor memory consolidation and regionally specific sleep spindles . PLoS One , 2 ( 4 ), e341 . 10.1371/journal.pone.0000341 17406665 PMC1828623

[b70] Oostenveld , R. , Fries , P. , Maris , E. , & Schoffelen , J. M. ( 2011 ). FieldTrip: Open source software for advanced analysis of MEG, EEG, and invasive electrophysiological data . Computational Intelligence and Neuroscience , 2011 ( 1 ), 156869 . 10.1155/2011/156869 21253357 PMC3021840

[b71] Peigneux , P. , Laureys , S. , Fuchs , S. , Collette , F. , Perrin , F. , Reggers , J. , Phillips , C. , Degueldre , C. , Del Fiore , G. , Aerts , J. , Luxen , A. , & Maquet , P. ( 2004 ). Are spatial memories strengthened in the human hippocampus during slow wave sleep ? Neuron , 44 ( 3 ), 535 – 545 . 10.1016/j.neuron.2004.10.007 15504332

[b72] Peirce , J. , Gray , J. R. , Simpson , S. , MacAskill , M. , Höchenberger , R. , Sogo , H. , Kastman , E. , & Lindeløv , J. K. ( 2019 ). PsychoPy2: Experiments in behavior made easy . Behavior Research Methods , 51 , 195 – 203 . 10.3758/s13428-018-01193-y 30734206 PMC6420413

[b73] Platel , H. , Baron , J. C. , Desgranges , B. , Bernard , F. , & Eustache , F. ( 2003 ). Semantic and episodic memory of music are subserved by distinct neural networks . NeuroImage , 20 ( 1 ), 244 – 256 . 10.1016/S1053-8119(03)00287-8 14527585

[b74] Qualtrics . ( 2005 ). Qualtrics . Provo, Utah , USA . https://www.qualtrics.com

[b75] R Core Team . ( 2018 ). R: A language and environment for statistical computing [Internet] (pp. 171 – 203 ). R Foundation for Statistical Computing .

[b76] Rakowska , M. , Abdellahi , M. E. , Bagrowska , P. , Navarrete , M. , & Lewis , P. A. ( 2021 ). Long term effects of cueing procedural memory reactivation during NREM sleep . NeuroImage , 244 , 118573 . 10.1016/j.neuroimage.2021.118573 34537384 PMC8591408

[b77] Rakowska , M. , Lazari , A. , Cercignani , M. , Bagrowska , P. , Johansen-Berg , H. , & Lewis , P. A. ( 2022 ). Distributed and gradual microstructure changes track the emergence of behavioural benefit from memory reactivation . BioRxiv . 10.1101/2022.04.28.489844 PMC12375995 40860580

[b78] Rasch , B. , Büchel , C. , Gais , S. , & Born , J. ( 2007 ). Odor cues during slow-wave sleep prompt declarative memory consolidation . Science , 315 ( 5817 ), 1426 – 1429 . 10.1126/science.1138581 17347444

[b79] Romano Bergstrom , J. C., Howard , Jr , J. H. , & Howard , D. V. ( 2012 ). Enhanced implicit sequence learning in college‐age video game players and musicians . Applied Cognitive Psychology , 26 ( 1 ), 91 – 96 . 10.1002/acp.1800

[b80] Sagi , Y. , Tavor , I. , Hofstetter , S. , Tzur-Moryosef , S. , Blumenfeld-Katzir , T. , & Assaf , Y. ( 2012 ). Learning in the fast lane: New insights into neuroplasticity . Neuron , 73 ( 6 ), 1195 – 1203 . 10.1016/j.neuron.2012.01.025 22445346

[b81] Schapiro , A. C. , McDevitt , E. A. , Rogers , T. T. , Mednick , S. C. , & Norman , K. A. ( 2018 ). Human hippocampal replay during rest prioritizes weakly learned information and predicts memory performance . Nature Communications , 9 ( 1 ), 3920 . 10.1038/s41467-018-06213-1 PMC6156217 30254219

[b82] Schmidt , D. , Krause , B. J. , Mottaghy , F. M. , Halsband , U. , Herzog , H. , Tellmann , L. , & Müller-Gärtner , H. W. ( 2002 ). Brain systems engaged in encoding and retrieval of word-pair associates independent of their imagery content or presentation modalities . Neuropsychologia , 40 ( 4 ), 457 – 470 . 10.1016/S0028-3932(01)00102-6 11684178

[b83] Schönauer , M. , Geisler , T. , & Gais , S. ( 2014 ). Strengthening procedural memories by reactivation in sleep . Journal of Cognitive Neuroscience , 26 ( 1 ), 143 – 153 . 10.1162/jocn_a_00471 23984946

[b84] Shadmehr , R. , & Holcomb , H. H. ( 1997 ). Neural correlates of motor memory consolidation . Science , 277 ( 5327 ), 821 – 825 . 10.1126/science.277.5327.821 9242612

[b85] Shanahan , L. K. , Gjorgieva , E. , Paller , K. A. , Kahnt , T. , & Gottfried , J. A. ( 2018 ). Odor-evoked category reactivation in human ventromedial prefrontal cortex during sleep promotes memory consolidation . Elife , 7 , e39681 . 10.7554/eLife.39681 30560782 PMC6298770

[b86] Tonegawa , S. , Morrissey , M. D. , & Kitamura , T. ( 2018 ). The role of engram cells in the systems consolidation of memory . Nature Reviews Neuroscience , 19 ( 8 ), 485 – 498 . 10.1038/s41583-018-0031-2 29970909

[b87] Trefler , A. , Sadeghi , N. , Thomas , A. G. , Pierpaoli , C. , Baker , C. I. , & Thomas , C. ( 2016 ). Impact of time-of-day on brain morphometric measures derived from T1-weighted magnetic resonance imaging . NeuroImage , 133 , 41 – 52 . 10.1016/j.neuroimage.2016.02.034 26921714 PMC5602560

[b88] van Dongen , E. V. , Takashima , A. , Barth , M. , Zapp , J. , Schad , L. R. , Paller , K. A. , & Fernández , G. ( 2012 ). Memory stabilization with targeted reactivation during human slow-wave sleep . Proceedings of the National Academy of Sciences of the United States of America , 109 ( 26 ), 10575 – 10580 . 10.1073/pnas.1201072109 22691500 PMC3387124

[b89] Veale , J. F. ( 2014 ). Edinburgh handedness inventory–short form: A revised version based on confirmatory factor analysis . Laterality: Asymmetries of Body, Brain and Cognition , 19 ( 2 ), 164 – 177 . 10.1080/1357650X.2013.783045 23659650

[b90] Wagner , A. D. , Shannon , B. J. , Kahn , I. , & Buckner , R. L. ( 2005 ). Parietal lobe contributions to episodic memory retrieval . Trends in Cognitive Sciences , 9 ( 9 ), 445 – 453 . 10.1016/j.tics.2005.07.001 16054861

[b91] Walker , M. P. ( 2005 ). A refined model of sleep and the time course of memory formation . Behavioral and Brain Sciences , 28 ( 1 ), 51 – 64 . 10.1017/S0140525X05000026 16047457

[b92] Walker , M. P. , Brakefield , T. , Hobson Allan , J., & Stickgold , R. ( 2003 ). Dissociable stages of human memory consolidation and reconsolidation . Nature , 425 ( 6958 ), 616 – 620 . 10.1038/nature01930 14534587

[b93] Walker , M. P. , Stickgold , R. , Alsop , D. , Gaab , N. , & Schlaug , G. ( 2005 ). Sleep-dependent motor memory plasticity in the human brain . Neuroscience , 133 ( 4 ), 911 – 917 . 10.1016/j.neuroscience.2005.04.007 15964485

[b94] Wenderoth , N. , Debaere , F. , Sunaert , S. , & Swinnen , S. P. ( 2005 ). The role of anterior cingulate cortex and precuneus in the coordination of motor behaviour . European Journal of Neuroscience , 22 ( 1 ), 235 – 246 . 10.1111/j.1460-9568.2005.04176.x 16029213

[b95] Wickham , H. ( 2009 ). ggplot2: Elegant graphics for data analysis . Springer . 10.1007/978-0-387-98141-3

[b96] Wilhelm , I. , Diekelmann , S. , Molzow , I. , Ayoub , A. , Mölle , M. , & Born , J. ( 2011 ). Sleep selectively enhances memory expected to be of future relevance . Journal of Neuroscience , 31 ( 5 ), 1563 – 1569 . 10.1523/JNEUROSCI.3575-10.2011 21289163 PMC6623736

[b97] Zhang , S. , & Li , C.-s.R. ( 2012 ). Functional connectivity mapping of the human precuneus by resting state fMRI . NeuroImage , 59 ( 4 ), 3548 – 3562 . 10.1016/j.neuroimage.2011.11.023 22116037 PMC3288461

