# Supplementary material for: Cueing memory reactivation during NREM sleep engenders long-term plasticity in both brain and behaviour
Source: Imaging Neurosci (Camb). 2024 Aug 1;2:imag-2-00250. doi: 10.1162/imag_a_00250 (PMC12272196; doi:10.1162/imag_a_00250)
Supplement: Supplementary Material [file imag_a_00250-supp.pdf]

Supplementary Materials for

Cueing memory reactivation during NREM sleep engenders long-term plasticity in both brain and behaviour.

Martyna Rakowska, Paulina Bagrowska, Alberto Lazari, Miguel Navarrete, Mahmoud E. A. Abdellahi,

Heidi Johansen-Berg, Penelope A. Lewis*

** Corresponding author: penlewis@gmail.com*

Supplementary Text

1. <u>Baseline SRTT performance</u>

Before sleep, no difference was found between the average reaction time of the cued and uncued sequence for either both hands (BH, $t_{29} = -0.25$, $p = 0.801$), left hand (LH, $t_{29} = 0.27$, $p = 0.786$) or right hand (RH, $t_{29} = -0.50$, $p = 0.621$) (paired-samples t-tests) dataset. Similar results were obtained where comparing random sequences before sleep for all datasets (BH: $z = -0.57$, $p = 0.572$; LH: $z = -0.63$, $p = 0.530$; Wilcoxon signed-rank tests; RH: $t_{29} = -0.16$, $p = 0.872$; paired-samples t-test). Thus, any post-sleep difference between the sequences can be regarded as the effect of TMR. Furthermore, average reaction times before sleep were significantly shorter for the last 4 sequence blocks than for the random blocks, confirming that the participants learned both sequences during S1 (BH cued: $z = -4.74$, BH uncued: $z = -4.49$; LH cued: $z = -4.78$, LH uncued: $z = -4.33$; $p < 0.001$ for all comparisons, Wilcoxon signed-rank test; RH cued: $t_{29} = 7.21$, RH uncued: 6.49; $p < 0.001$ for all comparisons, paired-samples t-test). Summary statistics for each sequence and dataset during S1 are presented in Table S1.

2. <u>Individual Hands Performance</u>

To unpack the effects of TMR and session on the SeqSpecS of LH and RH the same linear mixed effect model that we used for BH dataset (section 2.1.1) was fitted to the LH and RH datasets separately. TMR and session were entered as fixed effects, with participant specified as random effect. Results of all the likelihood ratio tests comparing the full model against the model without the fixed effect of interest are shown in Table S2B for LH and Table S2C for RH. Consistent with the analysis of BH trials, the analysis of LH and RH trials revealed a main effect of session on both early

(LH: $X^2(2) = 132.02$, $p < 0.001$, Table S2Bi; RH: $X^2(2) = 181.60$, $p < 0.001$, Table S2Ci) and late SeqSpecS (LH: $X^2(2) = 64.53$, $p < 0.001$, Table S2Bii; RH: $X^2(2) = 96.02$, $p < 0.001$, Table S2Cii). Post-hoc comparisons showed a difference between subsequent sessions (S2 vs S3, S3 vs S4) ($p_{adj} < 0.002$; Table S3B-C), suggesting continuous learning over time.

In line with our results for BH dataset, inclusion of TMR as a fixed effect improved model fit for the late SeqSpecS when each hand was analysed separately (LH: $X^2(1) = 4.02$, $p = 0.045$; Table S2Bii; RH: $X^2(1) = 15.46$, $p < 0.001$; Table S2Cii). Thus, the linear mixed effects analysis points to a main effect of TMR on the late SeqSpecS across all post-stimulation sessions and for each hand. Post-hoc comparisons performed on the RH dataset revealed a significant difference between the cued and uncued sequence performance at S4 ($p_{adj} = 0.006$) but not at S2 ($p_{adj} = 0.163$) or S3 ($p_{adj} = 0.119$) (Table S4C, Fig.S1A), consistent with our analysis of BH trials. However, the results differed for the LH dataset, where the TMR benefit at S4 ($p_{uncorr} = 0.040$) did not survive Holm correction (S4: $p_{adj} = 0.121$; S2: $p_{adj} = 0.421$; S3: $p_{adj} = 0.890$) (Table S4B, Fig.S1B). Together, these findings suggest that the difference between the cued and uncued sequence at 20 days post-TMR observed for BH dataset is driven by the dominant hand. Nevertheless, it is worth noting that although we do report a main effect of hand on the SeqSpecS (better performance for the dominant hand; $p < 0.001$), there was no interaction between hand and TMR ($p > 0.05$; Table S2D).

Finally, we explored how the cueing benefit evolves over time for each hand using a linear mixed effects analysis as before (section 2.1.2). Inclusion of the number of days post-TMR as the fixed effect improved model fit on the extent of cueing benefit only for the RH dataset ($\chi2(2) = 6.58$, $p = 0.010$; Fig.S1C), but not for LH dataset ($\chi2(2) = 0.74$, $p = 0.391$; Fig.S1D) (Table S5B-C). However,

there was neither a main effect of hand nor an interaction between hand and session ($p > 0.05$; Table S5D).

3. Explicit Memory Task

Given that TMR was shown to promote the emergence of explicit knowledge the next morning (McClelland et al., 1995), we also set out to test whether this is true after a longer period. However, we found no difference between the free recall of the cued and uncued sequence ($z = -0.568$, $p = 0.570$, Wilcoxon signed-rank test), suggesting no TMR effect on the explicit knowledge of the sequence 20 days post-encoding (Fig.S4). Nevertheless, performance on both sequences differed from chance (cued: $z = -4.14$, $p < 0.001$; uncued: $z = -4.29$, $p < 0.001$; Wilcoxon signed-rank test), indicating that the participants learned both sequences explicitly over the course of the experiment.

4. Questionnaires

The EHI confirmed that all participants were right-handed, as the laterality quotient score (ranging between -100 and +100, where the negative values indicate left-handers and positive right-handers) was +100% for all but one subject who scored +75%. PSQI global scores (on a 21-points scale) ranged between 1 and 7 points, with a mean of 3.67 ($\pm$0.28), indicating, on average, a 'good quality' of sleep (Deuker et al., 2013). The median answer to the SQ (with 1 and 9 indicating the highest and lowest level of alertness, respectively) was 2 (IQR: 1) for all sessions, suggesting similar levels of alertness throughout the study.

Participants did report hearing experimental sounds during the night: On a 3-points scale, the median answer was 2 (IQR: 2), with 33% of the participants not hearing any sounds (answer 1), 20%

of the participants being unsure (answer 2), and 47% of the participants hearing them clearly (answer 3). However, when asked about the number of sounds they had heard, the maximum number selected was 4 (reported by 13% of the participants), with the median answer of 2 (IQR: 2) sounds.

Supplementary Figures

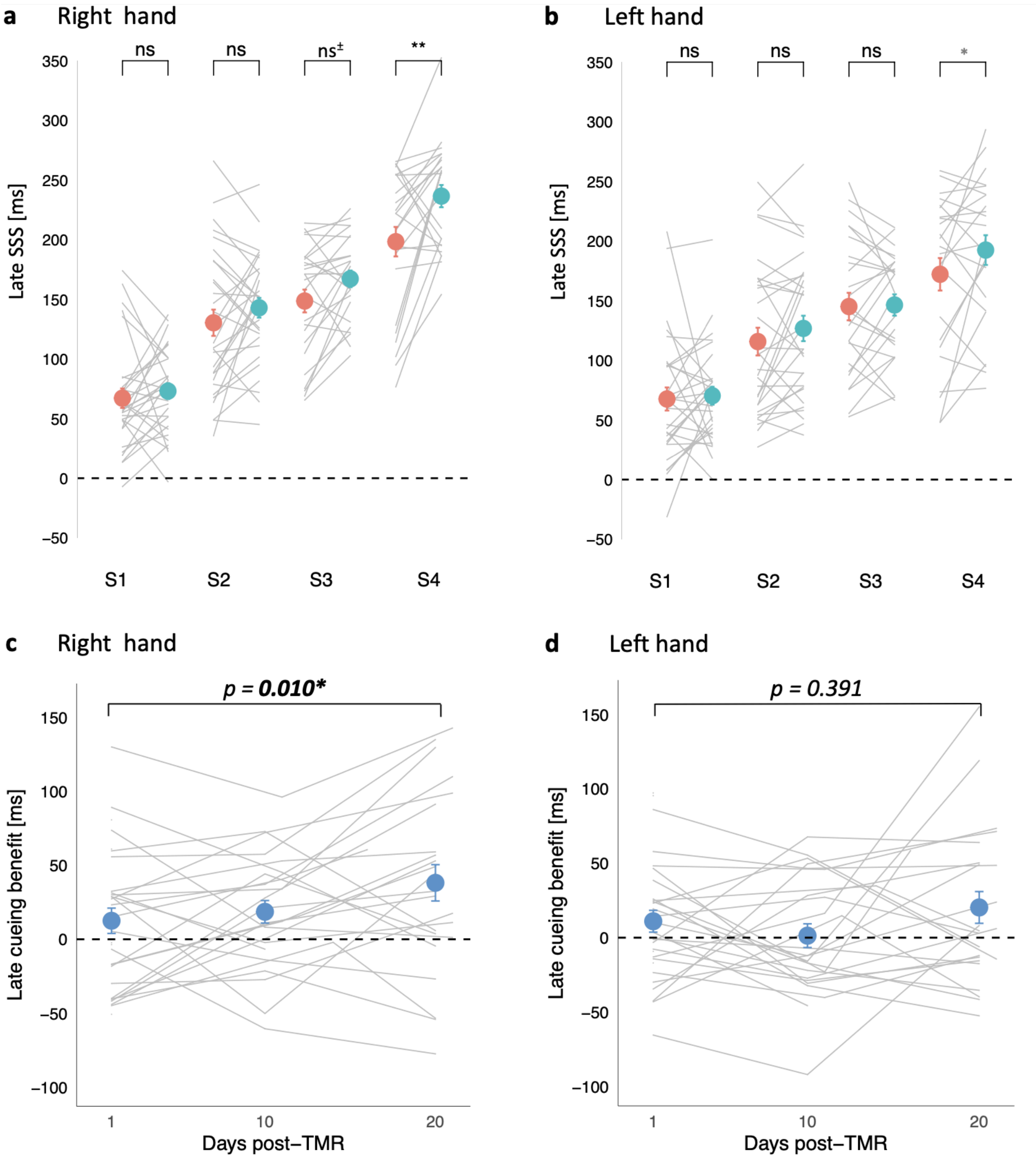

Fig. S1. Behavioural benefits of TMR are particularly strong for the dominant hand. (a-b) Mean late SeqSpecSfor the cued (blue dots) and uncued (red dots) sequence plotted against experimental sessions (S1-S4) for right-hand trials **(a)** and left-hand trials **(b)**. Error bars depict SEM. Grey lines represent individual participants. **(c-d)** Mean late SeqSpecS on the uncued sequence subtracted from the cued sequence for right

hand **(c)** and left hand **(d)**, plotted over time (number of days post-TMR). The effect of time was significant for the right-hand dataset only. Blue dots represent mean ±SEM calculated for S2, S3 and S4. Grey lines represent cueing benefit for each subject. For (a-d): n = 30 for S1-S2, n = 25 for S3, n = 24 for S4. S1-S4: Session 1 - Session 4; RT: reaction time; SeqSpecS: Sequence Specific Skill. **$p < 0.001$; *$p < 0.05$; ns$^{\pm}$: non-significant trend ($p = 0.060$); ns: non-significant; p-values shown in (a-b) are uncorrected; when adjusted for multiple comparisons using Holm's correction the effect of TMR at S4 remained significant only for (b) (black *) but not for (a) (grey *).

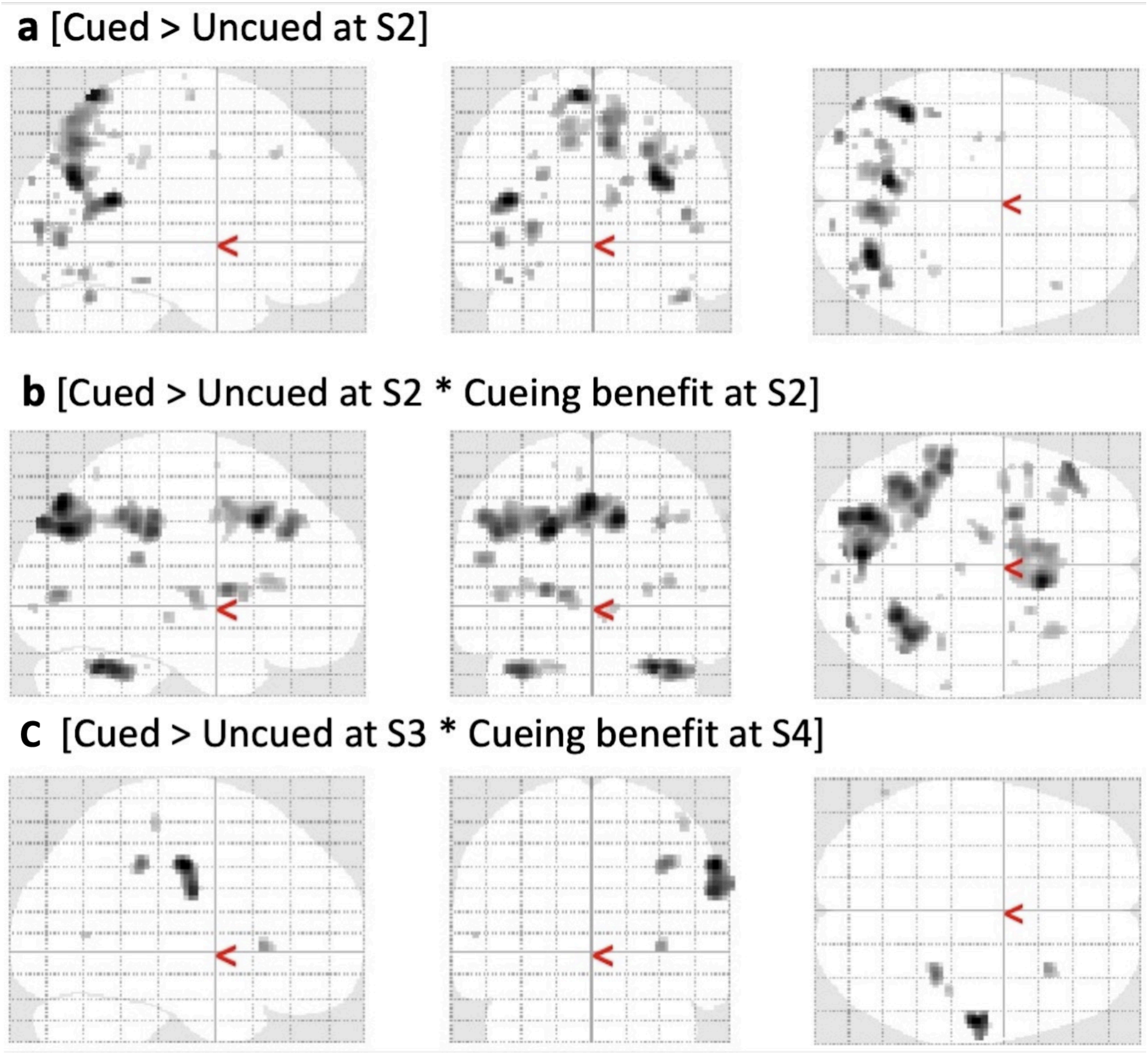

Fig. S2. Glass brain fMRI results. SPM fMRI results in glass brain projection displayed at $p < 0.001$, uncorrected, for the same contrasts as in Fig.4 and Fig.5A-B. **(A)** TMR-dependent increase in brain activity 24 h post-stimulation. **(B)** Brain activity for the cued > uncued contrast at S2 was positively associated with behavioural cueing benefit at the same time point. **(C)** Brain activity for the cued > uncued contrast at S3 was positively associated with behavioural cueing benefit at S4. S2-4: Session 2-4; n = 28 for (A-B), n = 21 for (C), n = 23 for (D).

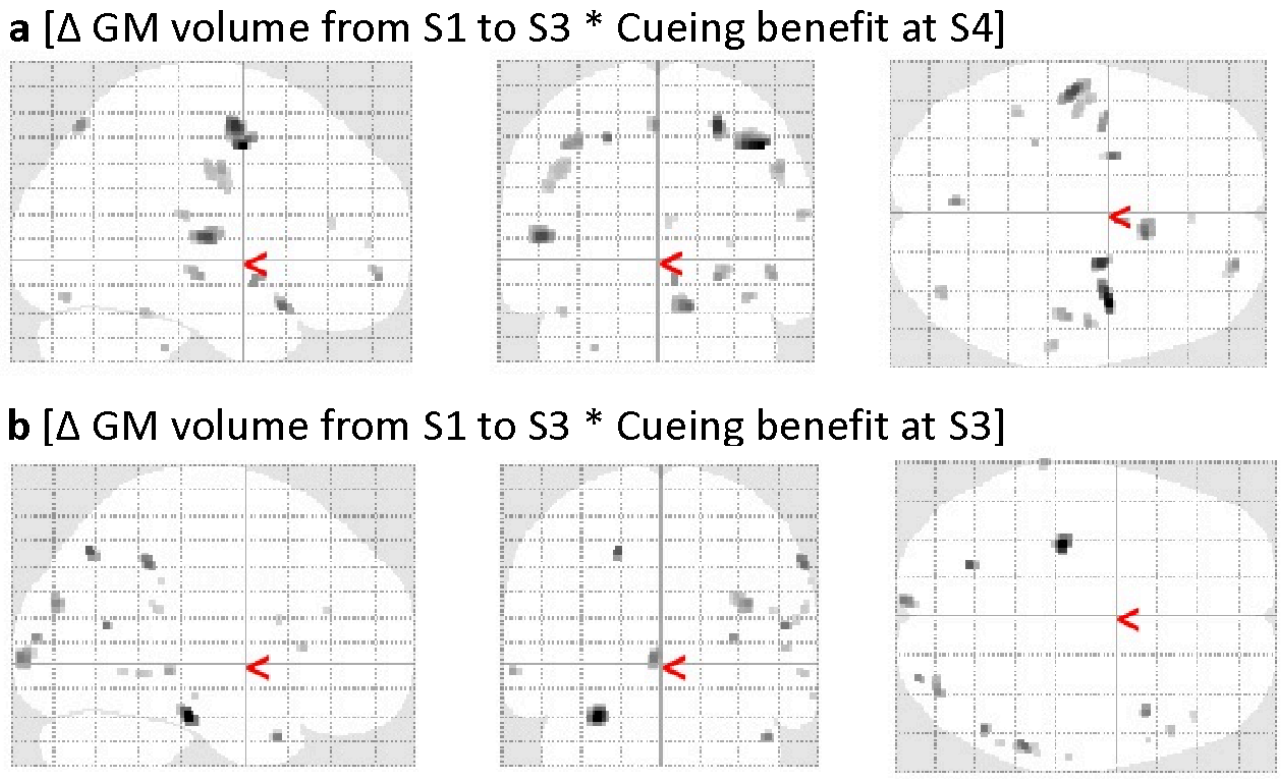

Fig. S3. Glass brain VBM results. SPM VBM results in glass brain projection displayed at $p < 0.001$, uncorrected, for the same contrasts as in Fig.5C-D (A) and Table S12 (A, B). **(A)** An increase in grey matter volume at S3 relative to S1 was associated with an increase in behavioural cueing benefit at S4. **(B)** Reduction in grey matter volume at S3 relative to S1 associated with cueing benefit at S3. S1-4: Session 1-4. n = 24 for (A), n = 29 for (B).

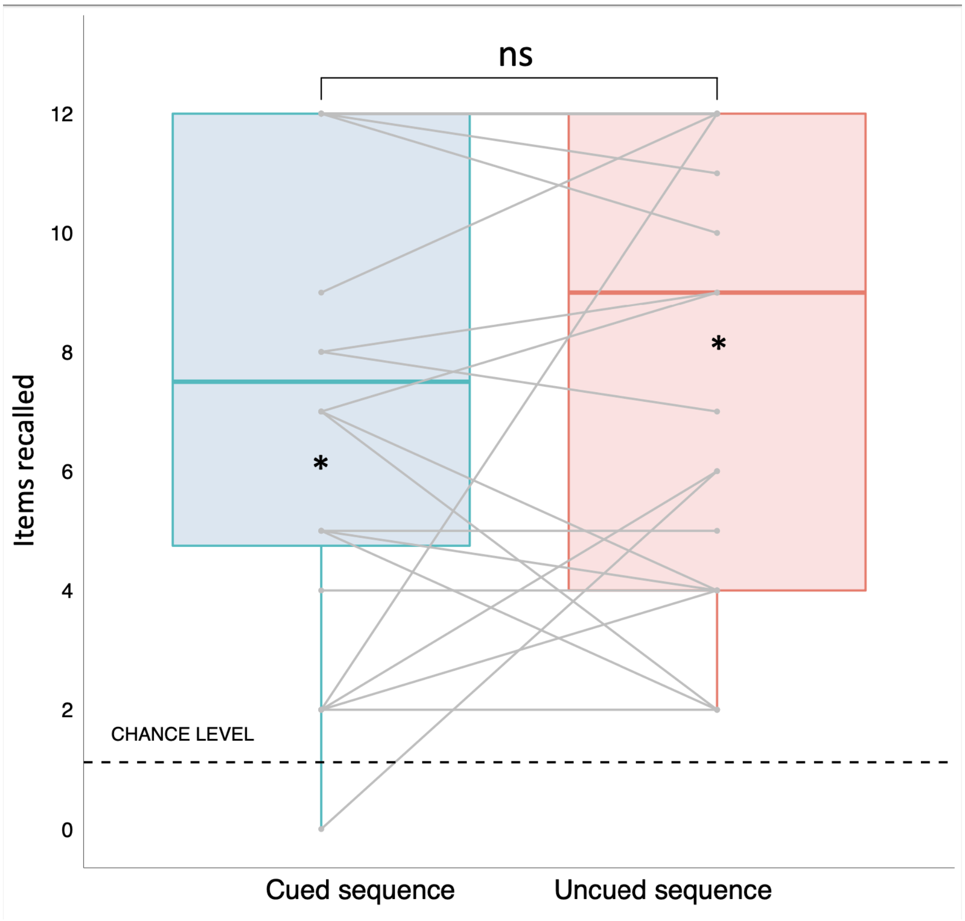

Fig. S4. Cueing memory reactivation during sleep does not affect explicit memory of the sequence. Explicit knowledge of both sequences was significantly above chance (significance denoted with *) 20 days post-encoding, although no effect of TMR was evident. Geoms represent median ±IQR for the cued (blue) and uncued (red) sequence, whiskers represent largest and lowest values within 1.5 IQR above and below the 75th and the 25th percentile, respectively. Grey dots represent performance of each subject. ns: non-significant. n = 24.

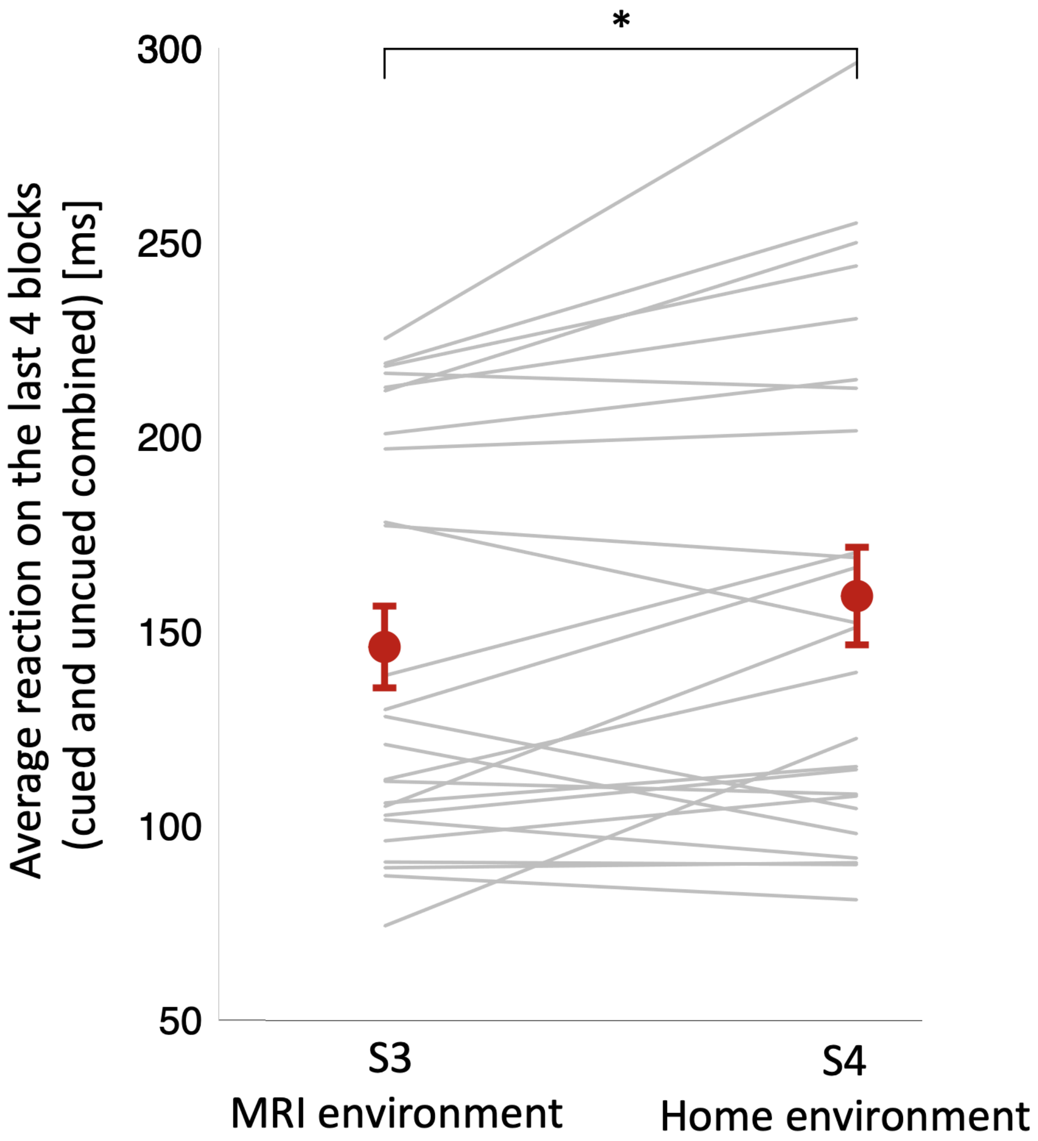

Fig. S5. Difference between the average reaction time on the last 4 blocks of S3 (performed in the MRI environment) compared to S4 (performed in home environment). Participants were significantly faster by the end of S3 than S4 (Paired t-test: t_{48} = -2.10, p = 0.041), suggesting that the MRI environment does not limit performance on the SRTT. Furthermore, we find no difference in variance between the two sessions (F test: F_{24} = 1.42, p = 0.200). * p < 0.05, n = 25.

Supplementary Tables

Table S1. SRTT summary statistics.

Mean reaction times (± SEM) (in ms) for the BH, LH and RH trials of the cued and uncued sequence blocks (24 blocks per sequence) as well as random blocks (2 blocks with tones matching the cued sequence and 2 blocks with tones matching the uncued sequence) during Session 1. Average reaction times (± SEM) for the last 4 blocks of each sequence are shown as well. BH: both hands; LH: left hand; RH: right hand. n = 30.

Dataset	Cued sequence	Uncued sequence	Cued random	Uncued random	Cued sequence (last 4 blocks)	Uncued sequence (last 4 blocks)
**BH**	321.82 ± 6.46	322.89 ± 7.83	357.81 ± 6.63	360.10 ± 6.69	286.05 ± 8.09	292.64 ± 9.99
**LH**	335.98 ± 7.18	334.90 ± 7.82	376.86 ± 7.45	371.60 ± 6.60	297.44 ± 9.19	303.92 ± 10.55
**RH**	307.67 ± 6.28	310.92 ± 8.44	347.77 ± 6.95	348.60 ± 7.76	274.62 ± 7.81	281.40 ± 10.20

Table S2. Effect of and interaction between TMR, hand and session.

Results of the likelihood ratio tests between the full, linear mixed effects model and reduced models, i.e., models without the fixed effect of interest, or with an interaction. The full model was used to test the effect of TMR, hand and session on the early and late SeqSpecS. df: degrees of freedom; $\chi 2$: chi-squared; AIC: Akaike Information Criterion; SeqSpecS: Sequence Specific Skill. *p < 0.05.

	df	χ2	p-value	AIC of a reduced model	AIC of a full model
**A. Both hands**					
i) Early SeqSpecS					
TMR	1	1.5450	0.2138	1632.4	1632.9
Session	2	175.77	**<0.0001***	1804.6	1632.9
TMR x Session	2	0.0740	0.9637	1636.3	1632.9
ii) Late SeqSpecS					
TMR	1	11.009	**0.0009***	1621.3	1612.3
Session	2	93.041	**<0.0001***	1701.3	1612.3
TMR x Session	2	3.0133	0.2216	1613.3	1612.3
**B. Left hand**					
i) Early SeqSpecS					
TMR	1	0.1878	0.6648	1666.5	1668.3
Session	2	132.02	**<0.0001***	1796.3	1668.3
TMR x Session	2	0.1044	0.9492	1672.2	1668.3
ii) Late SeqSpecS					
TMR	1	4.015	**0.0451***	1646.3	1644.2
Session	2	64.529	**<0.0001***	1704.8	1644.2
TMR x Session	2	1.9750	0.3725	1646.3	1644.2
**C. Right hand**					
i) Early SeqSpecS					
TMR	1	3.2309	0.0723	1651.0	1659.8
Session	2	181.60	**<0.0001***	1827.4	1649.8

TMR x Session	2	0.3741	0.8294	1653.4	1649.8
ii) Late SeqSpecS					
TMR	1	15.458	**<0.0001***	1636.8	1650.3
Session	2	96.024	**<0.0001***	1728.9	1636.2
TMR x Session	2	3.9605	0.1380	1636.9	1636.8
**D. Left and right hand combined**					
i) Early SeqSpecS					
Hand	1	22.423	**<0.0001***	3286.3	3265.9
Hand x Session	2	9.1678	**0.0102***	3260.7	3265.9
TMR x Hand	1	0.8955	0.3440	3267.0	3265.9
ii) Late SeqSpecS					
Hand	1	25.927	**<0.0001***	3261.5	3237.5
Hand x Session	2	6.4262	**0.0402***	3237.5	3235.9
TMR x Hand	1	2.1656	0.1411	3237.4	3237.5

Table S3. **Effect of session on SeqSpecS.**

Post-hoc pairwise comparisons between sessions for the early and late SeqSpecS, conducted on each dataset separately. P-values reported are Holm adjusted. SeqSpecS: Sequence Specific Skill; S2-4: Session 2-4; df: degrees of freedom. *p < 0.05.

	Mean S2 (±SE) [ms]	Mean S3 (±SE) [ms]	Mean S4 (±SE) [ms]	Estimate (±SE)	df	t ratio	p-value (Holm adj)	Effect size
**A. Both hands**								
i) Early SeqSpecS								
S2-S3	39.2 (9.88)	81.7 (10.20)	-	-42.5 (6.49)	135	-6.553	**<0.0001***	-1.320
S3-S4	-	81.7 (10.20)	163.1 (10.27)	-81.3 (6.63)	131	-12.270	**<0.0001***	-2.520
ii) Late SeqSpecS								
S2-S3	129.0 (8.92)	154.0 (9.24)	-	-24.7 (6.14)	135	-4.018	**0.0001***	-0.807
S3-S4	-	154.0 (9.24)	200 (9.30)	-46.2 (6.27)	131	-7.367	**<0.0001***	-1.513
**B. Left hand**								
i) Early SeqSpecS								
S2 – S3	35.3 (11.10)	73.3 (11.40)	-	-37.9 (7.34)	135	-5.227	**<0.0001***	-1.050
S3 – S4	-	73.3 (11.40)	144.1 (11.5)	-70.8 (7.50)	131	-9.553	**<0.0001***	-1.960
ii) Late SeqSpecS								
S2 – S3	121.0 (10.20)	149.0 (10.50)	-	-27.4 (6.74)	135	-4.064	**0.0001***	-0.817
S3 – S4	-	149.0 (10.50)	183 (10.6)	-34.4 (6.89)	131	-4.996	**<0.0001***	-1.026
**C. Right hand**								
i) Early SeqSpecS								
S2 – S3	43.0 (9.13)	90.0 (9.53)	-	-46.9 (7.13)	136	-6.627	**<0.0001***	-1.330
S3 – S4	-	90.0 (9.53)	182 (9.61)	-91.9 (7.30)	132	-12.670	**<0.0001***	-2.600
ii) Late SeqSpecS								
S2 – S3	137.0 (8.27)	159.0 (8.69)	-	-21.9 (6.90)	137	-3.167	**0.0019***	-0.634
S3 – S4	-	159.0 (8.69)	217 (8.77)	-58.1 (7.08)	132	-8.207	**<0.0001***	-1.685

Table S4. Effect of TMR on late SeqSpecS during each session.

Post-hoc pairwise comparisons of late SeqSpecS between the cued and uncued sequence on each session (S2-S4), conducted on each dataset separately. Both the uncorrected and Holm adjusted p-values are reported. S2-4: Session 2-4; SeqSpecS: Sequence Specific Skill; df: degrees of freedom. $^*p < 0.05$. $^\wedge p < 0.07$.

Cued vs Uncued	Mean cued (± SE) [ms]	Mean uncued (± SE) [ms]	Estimate (± SE)	df	t ratio	p value	p value (Holm adj)	Effect size
**A. Both hands**								
S2	135 (9.94)	123 (9.94)	-11.78 (7.95)	133	-1.482	0.1408	0.2815	-0.390
S3	157 (8.70)	147 (8.70)	-9.98 (8.71	133	-1.146	0.2537	0.2815	-0.331
S4	215 (11.60)	185 (11.60)	-29.13 (8.89)	133	-3.277	**0.0013***	**0.0040***	-0.965
**B. Left hand**								
S2	127 (11.20)	116 (11.20)	-11.02 (8.76)	133	-1.258	0.2106	0.4212	-0.3212
S3	146 (10.30)	145 (10.30)	-1.33 (9.60)	133	-0.139	0.8897	0.8897	-0.0401
S4	193 (13.10)	172 (13.10)	-20.29 (9.79)	133	-2.072	**0.0401***	0.1206	-0.6101
**C. Right hand**								
S2	143 (9.73)	130 (9.73)	-12.50 (8.94)	133	-1.401	0.1634	0.1634	-0.369
S3	167 (8.37)	149 (8.37)	-18.60 (9.79)	133	-1.899	0.0597^	0.1193	-0.548
S4	237 (11.00)	198 (11.00)	-38.20 (9.99)	133	-3.822	**0.0002***	**0.0006***	-1.125

Table S5. Effect of time and hand on the cueing benefit.

Results of the likelihood ratio tests between the full, linear mixed effects model and reduced models, i.e., models without the fixed effect of interest, or with an interaction. The full model was used to test the effect of hand and number of days post-TMR ('Time') on the cueing benefit (SeqSpecS for the uncued sequence subtracted from the cued sequence). $\chi 2$: chi-squared; AIC: Akaike Information Criterion. df: degrees of freedom. $^*p < 0.05$. $^\wedge p < 0.07$.

	df	χ2	p-value	AIC of a reduced model	AIC of a full model
**A. Both hands**					
Time	2	3.965	**0.046***	809.14	807.18
**B. Left hand**					
Time	2	0.736	0.391	825.46	826.73
**C. Right hand**					
Time	2	6.581	**0.010***	834.01	829.43
**D. Left and right hand combined**					
Hand	1	3.760	0.052^	1641.70	1639.90
Hand x Time	2	1.424	0.233	1639.9	1640.8

Table S6. Sleep parameters.

Total recording duration, total sleep time, time spent in each sleep stage and time scored as movement presented as average (minutes ± SEM) and as percentage of the total recording duration. Total sleep time was calculated by subtracting the time spent awake from the total recording duration. N1-N3: stage 1 – stage 3 of NREM sleep. REM: Rapid Eye Movement sleep. n = 29.

	Percentage of total recording duration [%]	Mean duration ± SEM [min]
Total recording duration	100%	524.19 ± 10.29
Total sleep time	88.38%	463.29 ± 12.89
Wake	11.50%	60.90 ± 10.37
N1	4.52%	23.33 ± 1.73
N2	46.35%	242.45 ± 8.41
N3	19.91%	104.22 ± 4.05
REM	15.86%	83.43 ± 4.30
Movement	1.61%	8.43 ± 1.32

Table S7. Cueing benefit and the duration of N2 and N3.

Results of Pearson's correlations between cueing benefit and the percentage of time spent in N2 and N3. Both the uncorrected and FDR corrected p-values are reported. df: degrees of freedom; S2-4: Session 2-4; SeqSpecS: Sequence Specific Skill; N2-3: Stage 2-3 of NREM sleep. *p < 0.05.

	Time spent in N2 [%]				Time spent in N3 [%]			
	df	Pearson's correlation	p-value	p-value (FDR corr)	df	Pearson's correlation	p-value	p-value (FDR corr)
S2	25	0.197	0.324	0.324	27	-0.015	0.939	0.939
S3	20	0.378	0.082	0.144	21	-0.031	0.887	0.939
S4	19	0.372	0.096	0.144	21	0.089	0.697	0.939

Table S8. Summary statistics for sleep spindles.

Average number and density (number/min) of spindles (± SEM). Results are presented for N2 and N3 of the cue (A) and no-cue (B) period. N2-3: stage 2-3 of NREM sleep.

	Density			Number		
	Average	Left	Right	Average	Left	Right
**A. Cue**						
N2	5.28 ±0.27	5.43 ±0.29	5.13 ±0.19	124.02 ±11.31	127.68 ±11.70	120.35 ±11.23
N3	4.24 ±0.16	4.31 ±0.16	4.17 ±0.16	191.58 ±13.14	195.39 ±13.88	187.77 ±12.74
N2 & N3	4.20 ±0.22	4.43 ±0.25	3.96 ±0.24	286.91 ±18.50	301.06 ±19.81	272.76 ±18.70
**B. No-Cue**						
N2	4.85 ±0.27	5.02 ±0.29	4.68 ±0.28	51.71 ±5.07	53.17 ±5.22	50.24 ±5.08
N3	3.86 ±0.16	3.94 ±0.17	3.78 ±0.17	79.48 ±5.68	81.30 ±5.79	77.66 ±5.69
N2 & N3	3.80 ±0.20	4.01 ±0.23	3.59 ±0.21	117.74 ±7.69	123.59 ±8.12	111.89 ±7.79

Table S9. Cueing benefit and spindle density.

Results of Pearson's (A) and Spearman's (B) correlations (both FDR corrected and not) between cueing benefit during each of the post-stimulation sessions (S2, S3, S4) and spindle density averaged over all motor channels during the cue (A) and no-cue (B) period. N2 and N3 were analysed separately and together (N23). df: degrees of freedom; S2-4: Session 2-4; N2-N3: stage 2 - stage 3 of NREM sleep.

Session	Sleep stage	Correlation coefficient	p-value	df	p-value (FDR corr)
**A. Cue period**					
S2	N2	0.237	0.224	26	0.288
S3	N2	-0.254	0.231	22	0.288
S4	N2	-0.231	0.288	21	0.288
S2	N3	-0.04	0.840	25	0.840
S3	N3	-0.337	0.126	20	0.192
S4	N3	-0.343	0.128	19	0.192
S2	N23	0.196	0.309	27	0.322
S3	N23	-0.277	0.191	22	0.322
S4	N23	-0.216	0.322	21	0.322
**B. No-cue period**					
S2	N2	0.135	0.485	27	0.485
S3	N2	-0.317	0.132	22	0.396
S4	N2	-0.227	0.296	21	0.444
S2	N3	0.227	0.235	27	0.546
S3	N3	-0.171	0.422	22	0.546
S4	N3	-0.132	0.546	21	0.546
S2	N23	0.189	0.326	27	0.639
S3	N23	-0.172	0.419	22	0.629
S4	N23	-0.042	0.851	21	0.851

Table S10. Functional TMR-related activity.

Clusters showing increased (inc) or decreased (dec) activity for the cued relative to uncued sequence alone (A - S2 and S3, B - S2) or when considering covariates of cueing benefit during S2 (C) and cueing benefit during S4 (D). No significant clusters were found when considering cueing benefit during S3 as a covariate.

Region	MNI x, y, z (mm)	Number of voxels	F/T peak	P_{FWE} peak
**A.** Main effect of TMR for S2 and S3				
i. Right Precuneus $_{(inc)}$	8, -72, 58	9	22.67	0.032
**B.** [Cued > Uncued at S2]				
i. Left Precuneus $_{(inc)}$	-9 -62, 66	9	4.79	0.020
**C.** [Cued > Uncued at S2 * cueing benefit at S2]				
i. Left Precuneus $_{(inc)}$	-4, -78, 46	40	5.18	0.009
ii. Left Precuneus $_{(inc)}$	-18, -68, 36	1	4.44	0.046
iv. Left Putamen $_{(inc)}$	-24, 4, 6	3	4.41	0.034
**D.** [Cued > Uncued at S3 * cueing benefit at S4]				
i. Right Postcentral gyrus $_{(inc)}$	58, -18, 38	7	5.50	0.022
ii. Left Parahippocampus $_{(dec)}$	-22, -26, -16	1	4.52	0.047

Regions listed were significant at peak voxel threshold of $p_{FWE} < 0.05$, after correction for multiple voxel-wise comparisons within pre-defined bilateral ROI for bilateral precuneus (A.i, B.i, C.i, C.ii), bilateral hippocampus and parahippocampus (E.ii), bilateral dorsal striatum (C.iii), bilateral sensorimotor cortex (D.i). Peak voxel MNI coordinates and peak F (A) and T (B-D) values are given. n = 22 for (A), n = 28 for (B) and (C), n = 23 for (D).

Table S11. MRI correlations with behavioural cueing effect.

Contrasts performed for the fMRI (A) and VBM (B) analysis, with columns denoting behavioural regressors (cueing benefit at S2, S3 and S4) and rows denoting the MRI images analysed. The contrasts marked with black ticks were performed to examine how functional and long-term structural changes in the brain could predict post-TMR behaviour improvements. Contrasts with neuroimaging data collected at baseline (S1) or where the neuroimaging data would predict past behavioural gains were not of interest. Significant findings (reported in Fig.4-5, Table S10, and Table S12) are marked with an asterisk and survived permutation analysis within 4 ROIs at a significance level of $p_{FWE} < 0.05$ (Table S13). S2-4: Session 2-4.

MRI images	Behavioural cueing benefit		
	S2	S3	S4
A. fMRI			
S2	✓* (precuneus)	✓	✓
S3		✓	✓* (postcentral gyrus)
B. VBM			
S1-S3		✓	✓* (precentral gyrus)

Table S12. Structural brain changes over time.

Clusters showing increased (inc) or decreased (dec) changes in grey matter volume over time and when considering covariates of cueing benefit during S3 and S4. No significant clusters were found when considering cueing benefit during S2 as a covariate. No significant clusters were found in white matter either.

Region	MNI x, y, z (mm)	Number of voxels	T peak	P_{FWE} peak
A. [GM volume from S1 to S3 * cueing benefit at S4]				
Right Precentral gyrus $_{(inc)}$	42, -2, 45	5	6.21	0.018
B. [GM volume from S1 to S3 * cueing benefit at S3]				
Left Fusiform $_{(dec)}$	-28, - 26, -24	5	5.51	0.025

Regions listed were significant at peak voxel threshold of $p_{FWE} < 0.05$, after correction for multiple voxel-wise comparisons within pre-defined ROI for bilateral sensorimotor cortex (A) and bilateral hippocampus and parahippocampus (B). Since fusiform gyrus was not our ROI, the result in (B) has likely arisen due to the imperfection of the method and is thus not discussed any further. Peak voxel MNI coordinates, and peak T values are given. n = 24 for (A), n = 29 for (B).

Table S13. Multiple ROIs correction for the correlational results reported in Fig. 5-6.

Results of the voxel-wise FWE corrected permutation analysis performed on each correlational result, correcting for all the ROIs.

Region	MNI x, y, z (mm)	Number of voxels before multiple ROI correction	Number of voxels	F peak	P_{FWE} peak
**A.** [Cued > Uncued at S2 * cueing benefit at S2]					
Left Precuneus $_{(inc)}$	-4, -78, 46	40	6	26.784	0.030
**B.** [Cued > Uncued at S3 * cueing benefit at S4]					
Right Postcentral gyrus $_{(inc)}$	58, -16, 38	7	3	30.215	0.041
**C.** [Δ GM volume from S1 to S3 * cueing benefit at S4]					
Right Precentral gyrus $_{(inc)}$	42, -2, 45	5	4	38.551	0.021

Regions listed were significant at peak voxel threshold of $p_{FWE} < 0.05$, after correction for multiple voxel-wise comparisons within a single mask combining all the pre-defined ROIs: (1) sensorimotor cortex, (2) hippocampus and parahippocampus, (3) dorsal striatum, (4) precuneus. Peak voxel MNI coordinates and peak F values are given. n = 28 for (A), n = 21 for (B), n = 24 for (C).

Supplementary References

Deuker, L., Olligs, J., Fell, J., Kranz, T. A., Mormann, F., Montag, C., Reuter, M., Elger, C. E., & Axmacher, N. (2013). Memory consolidation by replay of stimulus-specific neural activity. *Journal of Neuroscience*, 33(49), 19373-19383. doi:10.1523/JNEUROSCI.0414-13.2013

McClelland, J. L., McNaughton, B. L., & O'Reilly, R. C. (1995). Why there are complementary learning systems in the hippocampus and neocortex: insights from the successes and failures of connectionist models of learning and memory. *Psychological review*, 102(3), 419. doi:10.1037/0033-295X.102.3.419
